# Kidney-resident macrophages promote a proangiogenic environment in the normal and chronically ischemic mouse kidney

**DOI:** 10.1038/s41598-018-31887-4

**Published:** 2018-09-17

**Authors:** Amrutesh S. Puranik, Irina A. Leaf, Mark A. Jensen, Ahmad F. Hedayat, Ahmad Saad, Ki-Wook Kim, Abdulrahman M. Saadalla, John R. Woollard, Sonu Kashyap, Stephen C. Textor, Joseph P. Grande, Amir Lerman, Robert D. Simari, Gwendalyn J. Randolph, Jeremy S. Duffield, Lilach O. Lerman

**Affiliations:** 1 The Divisions of Nephrology & Hypertension, Mayo Clinic, Rochester, MN USA; 2 Rheumatology, Mayo Clinic, Rochester, MN USA; 3 Departments of Laboratory Medicine & Pathology, Mayo Clinic, Rochester, MN USA; 4 Departments of Immunology, Mayo Clinic, Rochester, MN USA; 5 Departments of Cardiovascular Diseases, Mayo Clinic, Rochester, MN USA; 6 University of Kansas, School of Medicine, Kansas City, KS USA; 7 University of Washington, Seattle, WA USA; 8 Vertex Pharmaceuticals, Boston, MA USA; 9 Biogen, Cambridge, MA USA; 10 Department of Pathology, Washington University School of Medicine, Saint Louis, MO USA; 11 Colton Center for Autoimmunity, New York University School of Medicine, New York, NY USA

## Abstract

Renal artery stenosis (RAS) caused by narrowing of arteries is characterized by microvascular damage. Macrophages are implicated in repair and injury, but the specific populations responsible for these divergent roles have not been identified. Here, we characterized murine kidney F4/80^+^CD64^+^ macrophages in three transcriptionally unique populations. Using fate-mapping and parabiosis studies, we demonstrate that CD11b/c^int^ are long-lived kidney-resident (KRM) while CD11c^hi^Mϕ, CD11c^lo^Mϕ are monocyte-derived macrophages. In a murine model of RAS, KRM self-renewed, while CD11c^hi^Mϕ and CD11c^lo^Mϕ increased significantly, which was associated with loss of peritubular capillaries. Replacing the native KRM with monocyte-derived KRM using liposomal clodronate and bone marrow transplantation followed by RAS, amplified loss of peritubular capillaries. To further elucidate the nature of interactions between KRM and peritubular endothelial cells, we performed RNA-sequencing on flow-sorted macrophages from Sham and RAS kidneys. KRM showed a prominent activation pattern in RAS with significant enrichment in reparative pathways, like angiogenesis and wound healing. In culture, KRM increased proliferation of renal peritubular endothelial cells implying direct pro-angiogenic properties. Human homologs of KRM identified as CD11b^int^CD11c^int^CD68^+^ increased in post-stenotic kidney biopsies from RAS patients compared to healthy human kidneys, and inversely correlated to kidney function. Thus, KRM may play protective roles in stenotic kidney injury through expansion and upregulation of pro-angiogenic pathways.

## Introduction

Renal artery stenosis (RAS) represents an increasingly common cause of ischemic chronic kidney diseases and irreversible kidney damage^1^. Failure to restore renal function in RAS is directly related to the extent of tissue injury^2^ and microvascular loss^3^. Cell-specific mechanisms like epithelial injury, infiltration of inflammatory monocytes, accumulation of macrophages, and dysregulation of developmental and innate immune pathways all play important roles in renal injury^4^.

Mononuclear-phagocytes orchestrate inflammation in the stenotic kidney^5,6^ and promote fibrosis. Macrophages exhibit phenotypic heterogeneity in response to tissue micro-environment, which may be determined partly by their cellular origins^7^. Circulating monocyte-derived macrophages arise from bone marrow (BM) progenitors, while tissue-resident macrophages (TRMϕ) are considered to originate from erythromyeloid progenitors during embryogenesis, and can self-renew autonomously in adult tissues, including the kidney^8^. In contrast to proinflammatory monocyte-derived macrophages, TRMϕ may participate in tissue repair, blunting fibrosis and inflammation^9^. To discern myleoid cells subtypes, the Immunological Genome Project has defined mouse dendritic cells (DCs), monocytes, and macrophages based on surface markers^10^. Co-expression of F4/80, CD64, MerTK, and FCRIV, is used to identify macrophages^10^ in the kidney^11^, lung, liver, spleen and gut^12^, where they prevent fibrosis by inducing tissue-specific repair programs.

In the mouse kidney F4/80^bright^ macrophages exhibit features of both DC and macrophages^13,14^. Phenotypic characterization of F4/80^bright^ macrophages, recently carried by Cao *et al*.^15^ and Stamatiades *et al*.^11^, suggest expression of MHCII, Cx3cr1, CD11c and FCRIV. Ontogeny studies by Ginoux and colleagues demonstrated that F4/80^bright^CD11b^int^ kidney macrophages derive from fetal monocytes that arise from erythromyleoid progenitors generated in the yolk sac^16^. Furthermore, parabiosis studies demonstrate that less than 1% of F4/80^bright^ cells exchange between parabiont mice and are Ccr2-independent^11^. Recent studies suggest functional role of kidney-resident macrophages (KRM) in recruiting monocytes and neutrophils in the kidney in response to small immune complexes. Furthermore, F4/80^bright^ KRM and the endothelial cells form a functional unit that monitors the transport of particles^11^, highlighting the physiological function of KRM. However, little is known about their role in RAS, an ischemic kidney disease marked by decrease of renal capillaries. Since KRM is the largest population of macrophages we sought to study its role in RAS.

In this study, based on the expression of CD11b and CD11c, we phenotypically classified renal F4/80^+^CD64^+^ macrophages in three subsets. Using parabiosis and fate-mapping, we identified KRM as F4/80^bright^CD11b^int^CD11c^int^ and monocyte-derived macrophages as CD11c^hi^Mϕ and CD11c^lo^Mϕ. All three macrophage populations expanded in the murine model of RAS, associated with loss of plasmalemma vesicle-associated protein (PLVAP)^+^CD31^+^ peritubular capillaries. Using irradiation followed by bone-marrow transplantation, we replaced native KRM with monocyte-derived KRM and studied the effect of RAS on monocyte-derived KRM. However, unlike the native KRM, monocyte-derived KRM did not sustain in RAS kidneys, and their loss was associated with amplified loss of peritubular capillaries. Adminsitration of liposomal clodronate too depleted native KRM which in turn resulted in loss of peri-tubular enodthelial cells. To explore the associated mechanisms we performed transcriptional profiling of all macrophages from Sham and RAS kidneys. We observed that native-KRM in RAS kidneys predominantly upregulate reparative pathways, like angiogenesis and wound healing. Furthermore, *in-vitro* studies demonstrated that co-incubation with RAS-KRM promote proliferation of peritubular endothelial cells. KRM-like CD11b^int^CD11c^int^CD68^+^ also increased in biopsies from human RAS kidneys compared to healthy subjects, and positively correlated with kidney function. Our findings suggest that KRM may protect the kidney during chronic ischemic injury.

## Results

### Renal macrophages comprise of long-lived KRM and monocyte-derived CD11c^hi^ and CD11c^lo^ macrophages

**Figure 1:**
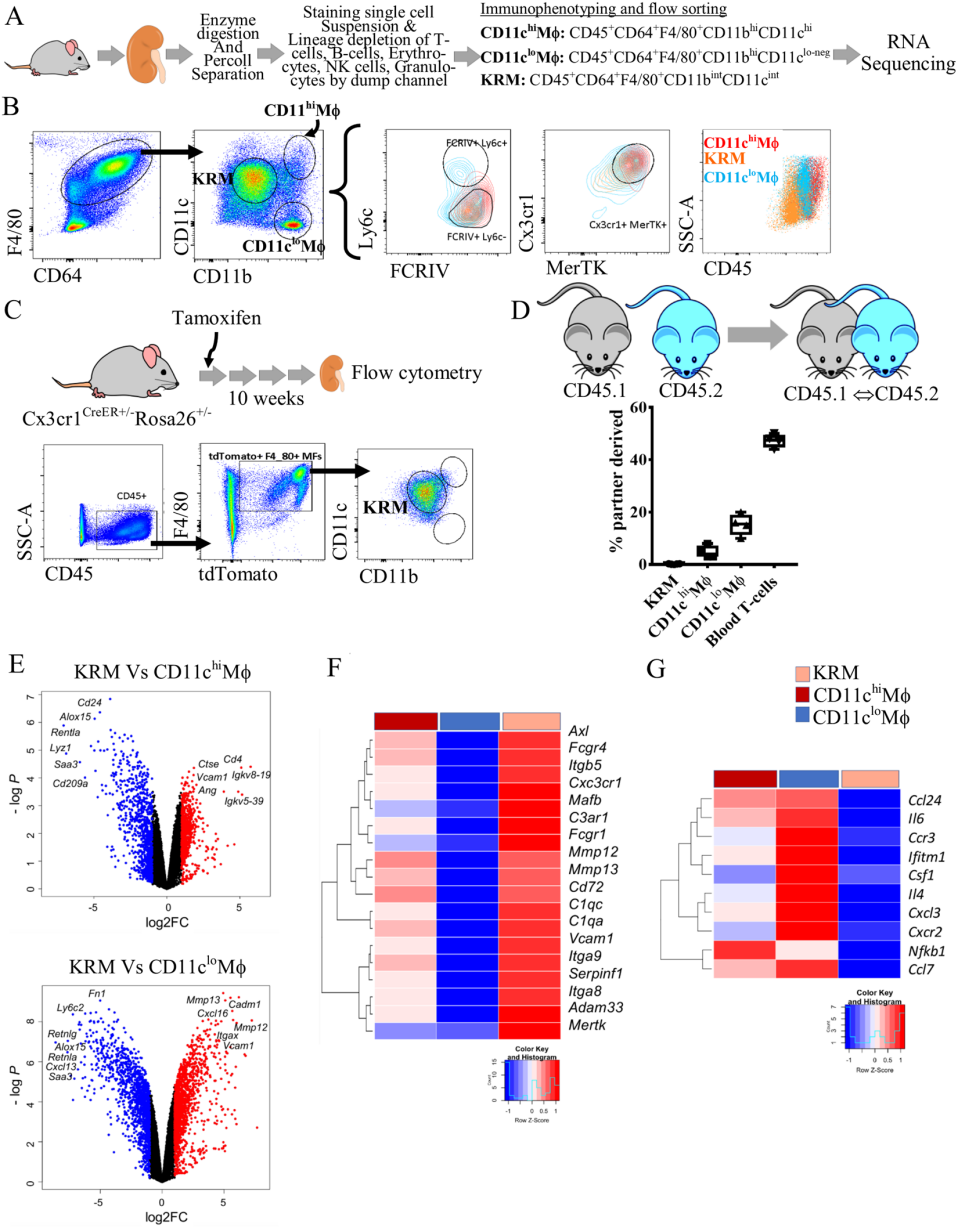
Renal macrophages comprise of long-lived kidney-resident macrophages and monocyte-derived CD11c^hi^ and CD11c^lo^ macrophages. **(A)** Workflow of the experiment. Mouse kidneys were enzyme-digested, percoll separated and stained for lineage and macrophage markers. After removing the lineage positive cells, three populations of macrophages were identified and flow sorted in the RNA lysis buffer and subjected to transcriptional profiling by RNA-sequencing. **(B)** Live, Lineage^neg^CD45^+^ were gated as F4/80^+^CD64^+/lo^ macrophages while non-macrophage population is CD45^+^11b/c^neg^CD64^neg^F4/80^neg^. We classified kidney macrophages as CD11c^hi^Mϕ (CD11b^hi^CD11c^hi^), CD11c^hi^Mϕ (CD11b^hi^CD11c^lo-neg^), and Kidney-resident macrophages (KRM) (CD11c^Int^CD11b^Int^). Overlay of CD11c^hi^Mϕ (red), CD11c^lo^Mϕ (blue) and KRM (orange) gated on Ly6c vs FCRIV, Cx3cr1 vs MerTK, and SSA vs CD45. KRM are Ly6c^−^FcrIV^+^MerTK^+^Cx3cr1^+^MHCII^+^CD45^int^ while the non-KRM CD11c^lo^Mϕ are FcrIV^+^MerTK^+^Cx3cr1^+^MHCII^+^Ly6c^hi^CD45^int-hi^ and CD11c^hi^Mϕ are FcrIV^+^MerTK^+^Cx3cr1^+^MHCII^+^Ly6c^lo^CD45^hi^. **(C)** Fate-mapping studies using Cx3cr1^CreER+/−^Rosa26^+/−^ mice demonstrates >80% of tdTomato^+^ cells gated as KRM. Live, Lineage^neg^CD45^+^ were gated as F4/80^+^tdTomato^+^ that were then gated as CD11b vs CD11c to identify KRM. **(D)** CD45.1 and CD45.2 congenic mice analyzed on 10 weeks of parabiosis. Histograms represents percentage of partner-derived cells in the kidney. n = 4 parabionts. Bars indicate mean value ± SEM. Symbols represent individual mice. **(E-G)** Transcriptional differences detected by RNA-Seq. **(E)** Comparison of gene expression between KRM and CD11c^hi^Mϕ (above), CD11c^lo^Mϕ (below) displayed as volcano plots of individual genes, where fold-change between populations is plotted on the x-axis and significance on the y-axis. Genes upregulated >2-fold are colored in red and genes downregulated >2-fold in blue. 1257 genes are differentially expressed between KRM and CD11c^hi^Mϕ, 649 are up- and 608 down-regulated; and 2674 genes are differentially expressed between KRM and CD11c^hi^Mϕ, 1386 are up- and 1288 down-regulated. **(F)** Selected genes reflecting tissue resident status and upregulated in KRM, and **(G)** inflammatory genes downregulated in KRM, are presented as heatmaps with hierarchical clustering. Mean values per macrophage populations are shown. The z-score-based color-scale shows gene expression standard deviations below (blue) or above (red) the population mean. Data is representative of n = 4 independent experiments with at least n = 3 mice per group (KRM, n = 4, CD11c^hi^Mϕ, n = 3, CD11c^lo^Mϕ, n = 3; DEGs: fold change >2, P < 0.05).Kidney image cropped and adopted from openclipart.org (https://openclipart.org/detail/28929/kidneyreins) and mouse images adopted from (https://openclipart.org/detail/174870/mouse and https://openclipart.org/detail/17558/simple-cartoon-mouse).

Cells were prepared by enzymatically digesting saline-perfused normal C57BL/6 mouse kidneys, followed by lineage depletion and antibody staining for macrophage markers (Figs 1A, S1A). To define the role of KRM in renal ischemia, we first identified F4/80^+^CD64^+/lo^ kidney macrophages by flow cytometry^12,17^. Using an imaging cytometer (FlowSight®, Millipore-Sigma) we confirmed that our macrophage gate consisted of both F4/80^Bright^ and F4/80^Dim^ populations that were positive for kidney macrophage marker FCRIV (Figs 1B, S1B, 2)^11^. Based on previous reports, we then considered CD11b^int^F4/80^bright^ kidney-resident macrophages and CD11b^hi^F4/80^+^ monocyte-derived macrophages (Fig. S1,B). We observed that kidney-resident macrophages were CD11c^int^ while monocyte-derived macrophages distinctly separated into CD11c^hi^ and CD11c^lo^ macrophages (Figs 1B, S1B). In summary, based on the expression of CD11b and CD11c we classified renal macrophages in three subsets, CD11b^hi^CD11c^hi^ (CD11c^hi^Mϕ), CD11b^hi^CD11c^lo-neg^ (CD11c^lo^Mϕ), and CD11b^int^CD11c^int^ subsequently considered as KRM (Fig. 1B, Table 1).

**Table 1:** Immunophenotype of renal cells identified and flow-sorted.

Cell Call	Phenotype	Ontogeny
CD11c^hi^Mϕ	CD45^hi^F4/80^+^CD64^+^CD11c^hi^CD11b^hi^FCRIV^+^Ly6c^int^CD43^+^	Non-classical monocyte-derived macrophages
CD11c^lo^Mϕ	CD45^hi^F4/80^+^CD64^+^CD11c^lo^CD11b^hi^FCRIV^+^Ly6c^hi^CD43^−^	Classical monocyte-derived macrophages
KRM	CD45^lo^F4/80^hi^CD64^+^CD11c^hi^CD11b^hi^FCRIV^+^Ly6c^−^CD43^+^	Kidney-resident macrophages

Furthermore, we performed phenotypic characterization on all three macrophage populations by flow cytometry. We observed that KRM were negative for Ly6c-a marker for blood derived cells, but expressed FCRIV, Cx3cr1, MHC class II and the lowest levels of CD45 (Figs 1B, S1B) as described previously^11,15,18^. Our observations agree with earlier studies suggesting that KRM account for >70% of macrophages^10,19,20^. Thus, KRM were further studied in detail. The CD11c^lo^Mϕ are Ly6c^hi^FCRIV^+^ and CD45^hi^, and therefore could represent classical monocyte-derived macrophages, while CD11c^hi^Mϕ are CD43^+^Ly6c^int^ and may be derived from non-classical monocytes^10,11,21^ (Table 1) (Figs 1B, S1B).

To define macrophage ontogeny, we performed fate-mapping and parabiosis studies. KRM were fate-mapped using a well-accepted model of Cx3cr1^CreER+/−^Rosa26^+/−^^22^. Upon tamoxifen injections most Cx3cr1 cells expressed tdTomato, but after 8–10 weeks the short-lived monocyte-derived cells lost tdTomato expression that only KRM retained. These tdTomato KRM were counted. We observed that >80% tdTomato^+^F4/80^+^ cells were CD11b^int^CD11c^int^ (Fig. 1C), suggesting that KRM are resident and long-lived, while both CD11c^hi^Mϕ and CD11c^lo^Mϕ are short-lived.

Similarly, analysis of parabiotic mice paired for up to 10 weeks showed that only <1% of KRM exchange between parabionts (Figs 1D, S4). Blood T-cells chimerism showed ~47% exchange (Figs 1D, S4). Among kidney macrophages, CD11c^lo^Mϕ displayed the highest exchange (15%) between parabionts, demonstrating their monocyte-derived origin. Interestingly, CD11c^hi^Mϕ showed ~5% exchange between parabionts, and since this population expresses CD43^+^, they may be non-classical monocyte-derived macrophages (Fig. S4).

To study transcriptional differences between these populations we flow sorted CD11c^hi^Mϕ, CD11c^lo^Mϕ and KRM in RNA lysis buffer (Qiagen) and subjected them to RNA-sequencing (Fig. 1A). Transcriptional expression was compared among all three macrophage populations (Fig. 1E–G). Principal component analysis (PCA) of macrophage RNA-sequencing showed separation between CD11c^hi^Mϕ, CD11c^lo^Mϕ and KRM in the second principal component with variance 24.31%. The CD11c^lo^Mϕ transcriptome appeared to be distinct from CD11c^hi^Mϕ and KRM. CD11c^hi^Mϕ and KRM were located closer in the PCA plot, and have more similar transcriptional profiles (Fig. S1E). In agreement with the PCA, we observed ~649 upregulated and ~608 downregulated genes in KRM vs. CD11c^hi^Mϕ (Fig. 1E). Genes upregulated >1.5 fold included known TRM markers like *Axl*, *Mafb*, *Cx3cr1*, *Vcam*, *Epor*, in addition to the microglial gene *Tmem119*. Genes downregulated in KRM and upregulated in CD11c^hi^Mϕ were peritoneal and red-pulp macrophage markers *Fcna*, *Lyz1*, *Alox15* and *Saa3*. We also observed *Cd209a* (DC-Sign) gene differentially expressed in CD11c^hi^Mϕ suggesting an overlap between DC and this macrophage population. Comparing KRM to CD11c^hi^Mϕ, ~1386 genes were upregulated and ~1288 down-regulated. Among upregulated in KRM were *Tmem119*, *Mmp12*, *Mmp13*, *Spp1*, C1qc and *C1qa* genes while down-regulated were *Fn1*, *Ly6c2*, *Retnlg*, *Retnla (*Fig. S11*)*.

The core macrophage signature genes identified in adult mice by the ImmGen consortium^10^, (*Cx3cr1*, *Mafb*, *FcgrIV*, *Mertk*, Axl, *Fcgr1*, *Csf1r*, *Spi1*, *Mafb*, *Myo7a*, *Tlr4*, and *Tlr7*) were indeed enriched in KRM compared to CD11c^hi^Mϕ and CD11c^lo^Mϕ (fold-change >1.5) (Figs 1F, S1D,E)^10^ confirming that flow cytometry-identified differences in cellular phenotypes can be reproducibly detected at the transcriptional level. Thus, the KRM that we identified are consistent with the postulated identity of long-lived, tissue-resident macrophages^21,23^.

Taken together, these data indicated that F4/80^bright^CD11b^int^CD11c^int^ are long-lived KRM, while infiltrating monocyte-derived macrophages are CD11c^hi^Mϕ and CD11c^lo^Mϕ. KRM are the largest population of macrophages in the healthy kidney, and we subsequently investigated their behavior pattern in chronic renal injury.

### KRM self-renew while monocyte-derived macrophages expand in ischemic kidneys correlating with loss of peri-tubular endothelial cells

**Figure 2:**
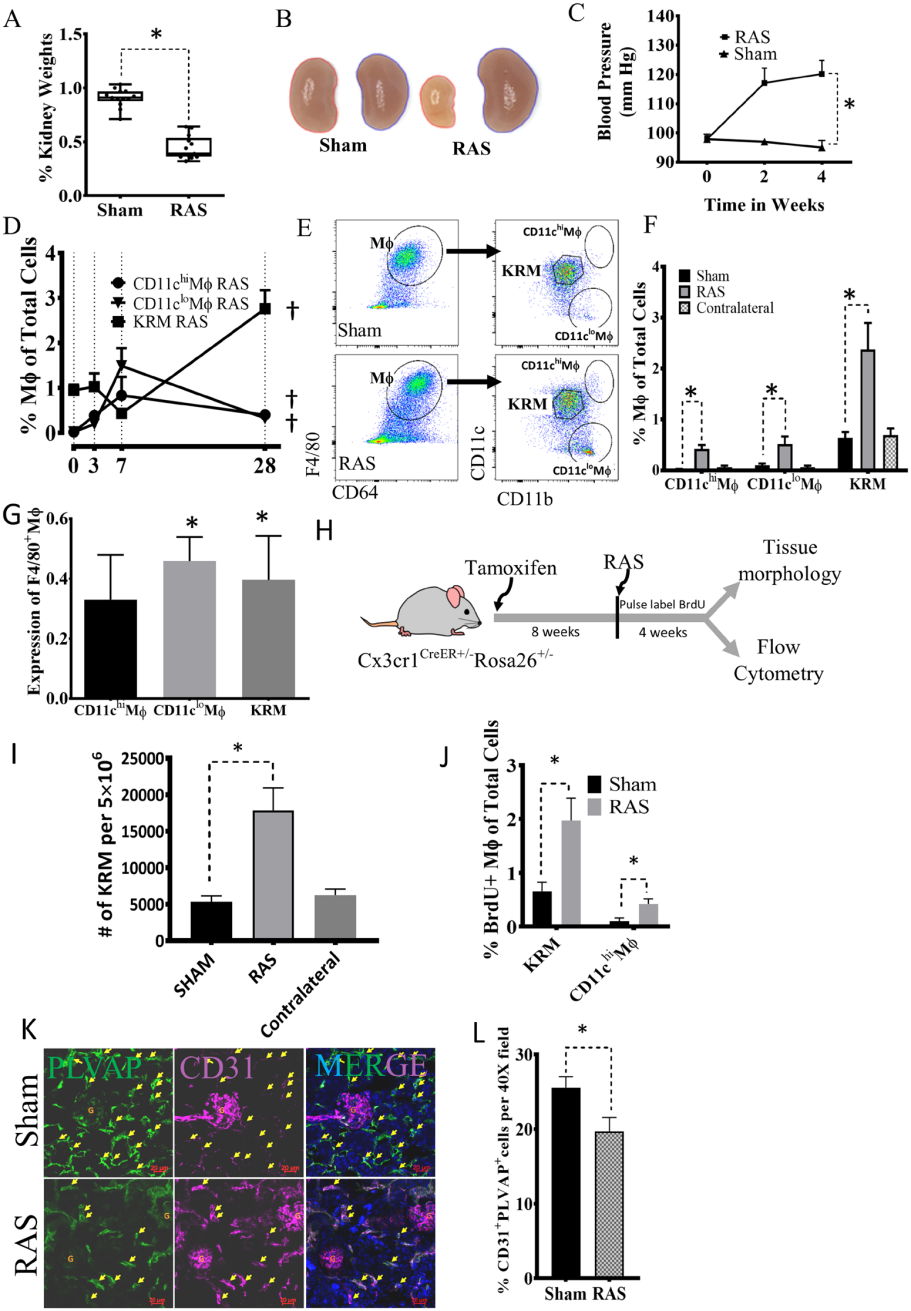
KRM self-renew while monocyte-derived macrophages expand in RAS kidneys and this increase correlates with loss of peri-tubular endothelial cells. **(A)** The ratio of stenotic relative to the contralateral kidney size falls after a RAS surgery (n = 20 sham and RAS mice). **(B)** Representative pictures show reduced size of stenotic (left) and increased size of contralateral (right) Sham and RAS kidneys. **(C)** Systolic blood pressure in RAS and Sham mice at weeks 0, 2, and 4. (**D)** CD11c^hi^Mϕ, CD11c^lo^Mϕ and KRM expand gradually in RAS kidneys, (n = 4–6/time point). **(E**, **F)** At day 28, RAS significantly increases stenotic kidney CD11c^hi^Mϕ, CD11c^lo^Mϕ and KRM compared to their respective Sham or contralateral kidney macrophages (n = 10–14). **(G)** Macrophage Expression (determined by resolution metric, see Supplemental Methods) significantly increases in CD11c^lo^Mϕ and KRM (n = 10–14). **(H)** Experimental Scheme for Fate-mapping of KRM Cx3cr1^CreER+/-^Rosa26^+/−^ mice. Tamoxifen was injected and after 8 weeks mice undergo RAS surgery. Mice were injected BrdU for 4 weeks and then euthanized. **(I)** Numbers of tdTomato + ve KRM in Cx3cr1^CreER+/−^ mice significantly increase with RAS compared to Sham (n = 5 each). **(J)** BrdU-positive KRM and CD11c^hi^Mϕ in RAS and Sham, indicating proliferation. **(K**, **L)** Immunofluorescence quantification of peri-tubular endothelial cells by co-staining for CD31 and PLVAP in Sham and RAS. ^†^P < 0.05 vs Day-0 of the same group; *P < 0.01 vs Sham. Kidney pictures taken by AP.

RAS caused hypertension and progressive loss of stenotic kidney volume (Figs 2A–C, S5A)^24^. Flow cytometric analysis showed increased numbers of total CD11b^+^F4/80^+^ macrophages and complementary DC (cDC1) in the stenotic kidney after 4 weeks of RAS (Fig. S5,A–C). Furthermore, the number of CD11c^hi^Mϕ, CD11c^lo^Mϕ, and KRM steadily rose with duration of ischemia (Fig. 2D). By 4 weeks the number and expression (Fig. 2E–G) of all macrophages increased in stenotic compared to Sham and contralateral kidneys, suggesting that both resident and monocyte-derived macrophages respond to ischemic injury. The CD11c^lo^Mϕ subset was Ly6C^hi^, while CD11c^hi^Mϕ and KRM were Ly6C^lo^. The increase in CD11c^hi^Mϕ and CD11c^lo^Mϕ in stenotic kidneys resulted from recruited Ly6C^hi^ monocytes differentiating into Ly6C^hi^ Mϕ that contribute to CD11c^lo^Mϕ, or become Ly6C^lo^ macrophages that contribute to CD11c^hi^Mϕ, both of which are CD11b^hi^, in agreement with previous studies in unilateral ureteral obstruction^25^. CD11b^hi^ cells promote fibrosis and macrophage infiltration in injured kidneys^26,27^. Thus, our data are consistent with the notion that CD11c^hi^Mϕ and CD11c^lo^Mϕ in the stenotic kidney in part differentiate from recruited Ly6C^hi^ monocytes and might cause inflammatory kidney damage.

Using CX3CR1^creER^:Rosa26-tdTomato reporter mice, we then fate-mapped and studied KRM kinetics in RAS (Fig. 2H). KRM number increased in RAS (Figs 2I, S6A), and BrdU pulse-labeling demonstrated that KRM and CD11c^hi^Mϕ expanded (Figs 2J, S6B). Interestingly, a small fraction of BrdU^+^CD11b^int^Mϕ was tdTomato^neg^, suggesting that in RAS some infiltrating monocytes may contribute to KRM. Thus, all macrophages increase in response to renal ischemia, associated with renal fibrosis (Trichrome and Picrosirius-red staining) (Fig. S5F) and capillary loss (Fig. 2K,L). Peritubular microvascular loss was ascertained using immunofluorescence of CD31 and PLVAP (Fig. 2K), which selectively stains peritubular capillaries^28^, and flow cytometry confirmed reduced PLVAP^+^CD31^+^ cells in stenotic kidneys (Fig. S5D), suggesting that RAS induces capillary rarefaction. This was associated with increased expression of pro-inflammatory genes (Fig. S5E) and fibrosis (Fig. S5F) in RAS kidneys. Thus, in renal ischemia monocyte-derived macrophages are recruited from the circulation, while KRM are long-lived and progressively self-renew. The overall increase in macrophages is associated with loss of peritubular capillaries and renal fibrosis.

### Donor-derived monocytes repopulate the KRM niche

**Figure 3:**
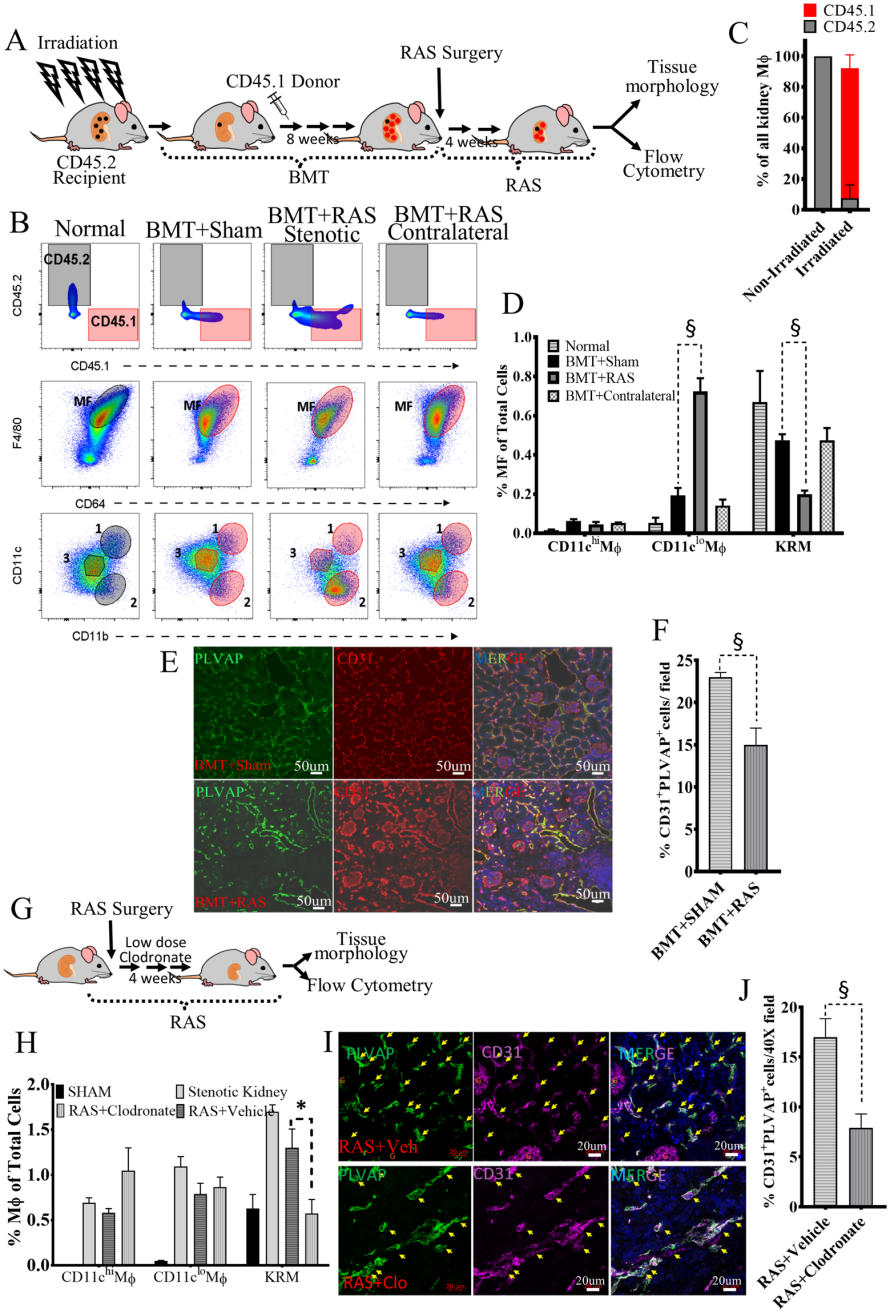
KRM repopulate from donor bone marrow (BM) cells but do not sustain in stenotic kidneys resulting in loss of peritubular capillaries. **(A)** Experimental Schema for BM transplantation (BMT) studies. **(B**–**D)** Reconstitution by donor-derived (CD45.1) BM in BMT + Sham, BMT + RAS stenotic and BMT + RAS contralateral kidneys (Top Row); Native (black tinted) and donor-derived macrophages (pink tinted) (Middle Row); Donor-derived KRM population decreased in BMT + RAS (Bottom Row). **(E)** Immunostaining of PLVAP and CD31 showing peri-tubular endothelial cells in representative images of BMT + Sham and BMT + RAS kidney sections. Images acquired on Zeiss confocal at 40X and stitched together to show a larger area. Note CD31 (red) stains peri-glomerular cells while PLVAP (green) is specific to peri-tubular endothelial cells. **(F)** Quantifying PLVAP^+^CD31^+^ cells showing significant reduction in BMT + RAS Vs BMT + Sham. **(G)** Experimental Schema for administration of liposomal clodronate at low-doses. **(H)** Comparing percent of all three macrophages in the stenotic kidneys of Sham, RAS, RAS + Vehicle and RAS + Clodronate mice. Note significant reduction in KRM in RAS + Clodronate group. **(I)** Immunostaining of PLVAP and CD31 showing peri-tubular endothelial cells in representative images of RAS + Vehicle and RAS + Clodronate kidney sections. **(J)** Quantifying PLVAP^+^CD31^+^ cells. Significant loss of peritubular endothelial cells seen after administration of clodronate. n = 6 mice/group; *P ≤ 0.01 vs Sham; ^§^P < 0.05 vs BMT + Sham , RAS + Vehicle; ^¥^P < 0.01 vs RAS. Mouse images adopted from Openclipart.org https://openclipart.org/detail/174870/mouse and https://openclipart.org/detail/28929/kidneyreins.

To test whether KRM repopulate from BM, we irradiated wild-type C57BL/6 mice (CD45.2), and transplanted bone marrow (BMT) from CD45.1 mice (Fig. 3A). All three renal macrophage populations were replenished by donor-derived BM (Fig. 3B,C). Thus, under stress conditions, monocytes repopulate the TRM niches in an attempt to restore KRM^29^. In the liver, circulating monocytes completely populate empty Kupffer cell niches and eventually form fully functional monocyte-derived Kupffer cells. Similarly, monocyte-derived alveolar macrophages demonstrate similar gene expression profile as embryonic alveolar macrophages^30,31^. Thus, BM cells can repopulate the KRM niche in the irradiated non-stenotic kidneys.

### Depletion of KRM amplifies RAS-associated microvascular rarefaction

To assess the role of KRM in microvascular rarefaction we used two strategies. First, native KRM were replaced by donor-derived KRM in BMT followed by RAS induction in mice (Fig. 3A) and secondly, continuous depletion of native KRM was induced using low-doses of liposomal clodronate in Sham and RAS mice (Fig. 3G).

Interestingly, donor-derived KRM diminished in BMT stenotic kidneys after 28d of RAS (Fig. 3B,D), contrasting with the increase observed in non-irradiated RAS kidneys (Fig. 2D). Donor-derived CD11c^hi^Mϕ numbers also decreased, whereas CD11c^lo^Mϕ numbers increased. Contralateral and sham kidneys remained unchanged (Fig. 3D). Fibrosis was not different in BMT + RAS compared to RAS kidneys (Fig. S7A,B), whereas the loss of PLVAP^+^CD31^+^ peri-tubular endothelial cells was amplified (Fig. 3E,F). Thus, donor-derived KRM repopulate native KRM niches, but do not replicate their pro-angiogenic properties thereby amplifying RAS-associate capillary loss.

Previous studies have administered higher doses of clodronate multiple times to achieve complete macrophage depletion^32–34^. In our model, liposomal clodronate at a single intraperitoneal dose of 200 ul significantly reduced blood monocytes (Fig. S8A), yet did not completely deplete KRM (Fig. S8B). Also, the depleted macrophages were replenished within 72 hours (Fig. S8B). Infiltrating monocytes and the remaining native KRM may have replenished the macrophage pool. Moreover, depletion of macrophages provoked neutrophil influx. To achieve gradual and continued depletion of KRM, and restrict neutrophil influx, we therefore subsequently used low-doses of clodronate 100 ul (FormuMax, Scientific CA) intraperitoneally every 4 days for 4 weeks.

Administration of liposomal clodronate to RAS mice selectively reduced KRM, but not the CD11c^lo^Mϕ and CD11c^hi^Mϕ (Fig. 3H, Fig. S8D). Reduction of KRM was associated with reduced number of PLVAP^+^CD31^+^ cells (Fig.I, J) and increased fibrosis (Fig. S8F) in the stenotic kidneys of the RAS + clodronate group. Similarly, the expression of anti-inflammatory and pro-angiogenic genes such as Arg1, Il4, Il10, Smad7, Angpt1, Igf1, Vcam1, Agtr2, Stat6, Mertk, Icam1 and the transcription factor Hbp1 (Fig. S8G,H) was reduced in KRM from RAS + clodronate group. These findings suggested that monocyte-derived cells could replenish KRM, but in the pathological setting of renal ischemia the monocyte-derived KRM lack the reparative ability of native KRM.

Menezes *et al*. observed that monocyte-derived Kupffer cells repopulated the liver to give rise to kupffer cells, but were unable to perform native kupffer cell functions^31^. Monocyte-derived alveolar macrophages express significantly higher pro-fibrotic genes than native embryonic-derived alveolar macrophages in bleomycin induced lung injury^35^. Thus, in active disease the monocyte-derived KRM may be less reparative than native KRM.

### Native KRM respond to RAS by upregulating pro-angiogenic and reparative genes

**Figure 4:**
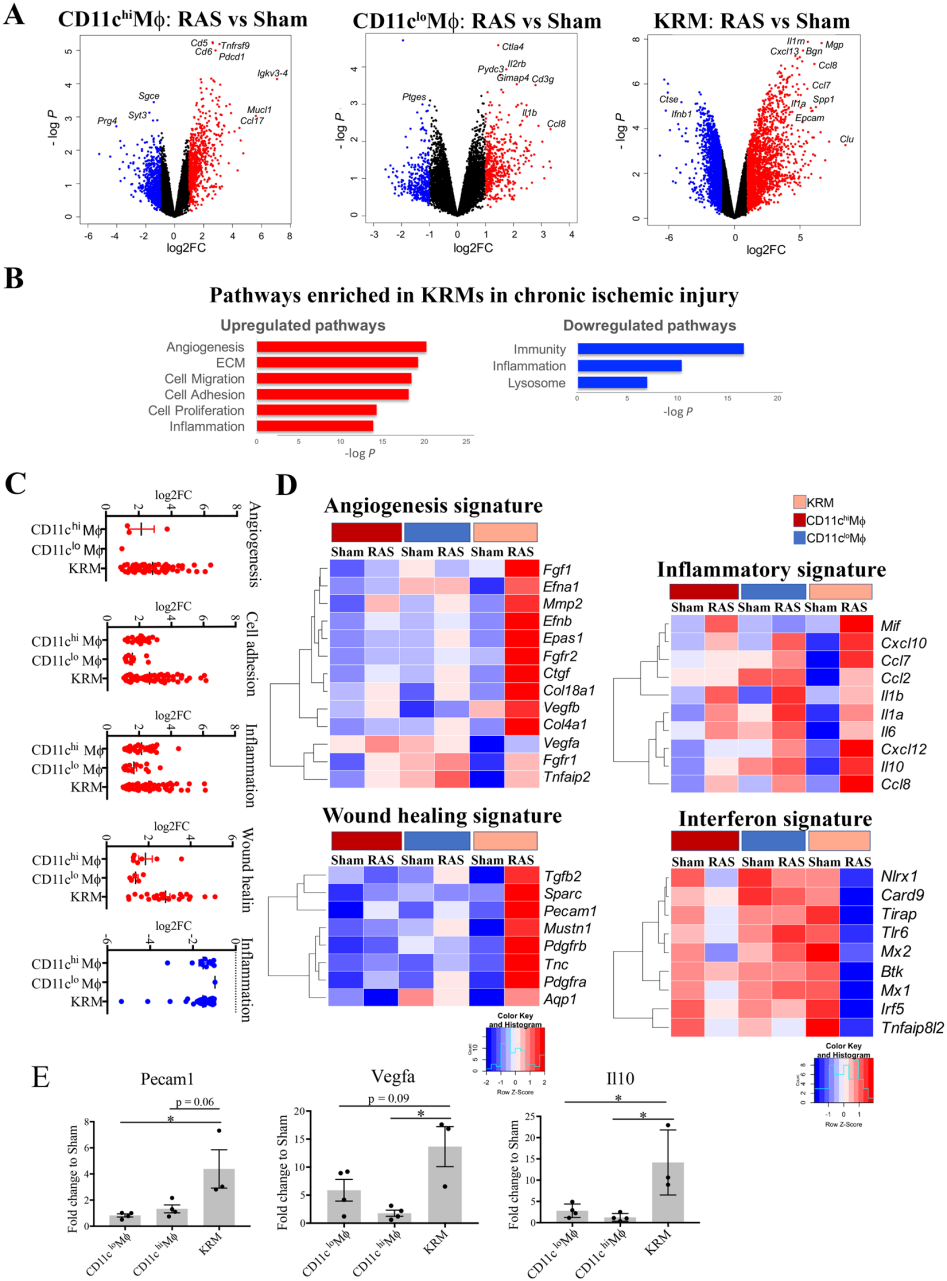
Transcriptional profiling of Kidney-resident macrophages (KRM) demonstrates upregulation of Angiogenesis and Wound healing pathways in stenotic kidneys. **(A)** Ischemia-associated gene expression changes in RAS compared to Sham kidneys in CD11c^hi^Mϕ, CD11c^lo^Mϕ and KRM, respectively, displayed as volcano plots of individual genes, where fold-change between populations is plotted on the x-axis and significance on the y-axis. Genes upregulated >2-fold are in red, and genes downregulated >2-fold in blue. In RAS-CD11c^hi^Mϕ, 934 genes show changes in expression, with 766 up- and 168 down-regulated; while in RAS- CD11c^lo^Mϕ, 307 genes change, 241 up- and 66 down-regulated; and KRMs display the greatest changes in stenotic injury, with 3162 DEGs; 1506 are up- and 1656 down-regulated. **(B)** Enrichment analysis of biological process ontology in CD11c^hi^Mϕ, CD11c^lo^Mϕ and KRM. Top upregulated (top, red) and downregulated (bottom, blue) pathways in macrophage populations isolated from stenotic compared to Sham kidneys (pathway enrichment P < 0.05). **(C)** Contributions of different macrophage populations to injury response shown as log2 fold-change in expression of CD11c^hi^Mϕ, CD11c^lo^Mϕ and KRM. Each dot represents a gene that is up (red) or downregulated (blue) in RAS > 2-fold compared to sham control. **(D)** Gene signatures that are upregulated (angiogenesis, wound healing, and inflammation) or downregulated (interferon signature) in KRM in injury presented as heat-maps with hierarchical clustering. Mean values per Mϕ populations are shown. The z-score-based color-scale shows gene expression standard deviations below (blue) or above (red) the population mean. **(E)** Expression of genes involved in angiogenesis, and anti-inflammatory response presented as fold change over respective Sham and validated by RT-qPCR for individual macrophage samples from Sham and RAS mice. RAS KRM n = 3, RAS CD11c^hi^Mϕ, n = 3 and RAS CD11c^lo^Mϕ, n = 3; ^*^P < 0.01 RAS-KRM vs RAS-CD11c^hi^Mϕ and *P < 0.01 RAS-KRM vs RAS-CD11c^lo^Mϕ. #P < 0.01 RAS vs respective Sham.

To examine the effect of renal ischemia on kidney macrophage transcriptome, we flow-sorted macrophages, studied their gene expression by RNA-seq, and validated upregulated genes by TaqMan Low-Density Arrays. A volcano plot of differentially expressed genes (DEGs) demonstrates significant changes in KRM isolated from RAS kidneys compared to sham (Fig. 4A). We studied gene ontology and functional network analysis of DEGs using DAVID and GORILLA. Stenotic-kidney monocyte-derived CD11c^hi^Mϕ and CD11c^lo^Mϕ upregulated pro-inflammatory pathways, like response to LPS (GO:0032496; p = 1.6E-06) and NFKB signaling pathway (mmu04064, p = 2.1E-06). Contrarily, RAS-KRM presented reparative pathways like wound healing (GO:0042060; p = 3.81E-09), angiogenesis (GO:0001525; p = 3.2E-21), and positive regulation of endothelial cell proliferation (GO:0001938; p = 7.8E-07) (Fig. 4B–E), while downregulating immune pathways like complement activation (GO:006958) and response to bacterium (GO:0042742). Angiogenesis pathway includes 62 genes such as *Vegfa*, *Lcn2*, *Agtr2*, *Angpt1*, and *Vcam1*. Vegf can act through *Lnc2* and induces proliferation in endothelial cells^36^ (Fig. 4C,D). Similarly, Vegf-expressing macrophages facilitate liver repair after ischemic injury. Upregulated genes also include *Spp1* and *Ccl2*. Indeed, osteopontin (Spp1) triggers macrophages to secrete *Ccl2*, which in turn promotes growth of adjacent endothelial cells^37^. Of these 62 genes *Vegfa*, *Vegfb*, *Pecam1*, *Pdgfrb*, *Tnc*, *Ctgf*, and *Aqp1* also contribute to wound healing, underscoring the reparative transcriptional changes in KRM in RAS (Fig. 4C–E).

Wound healing pathways upregulated by RAS-KRM included classical tissue repair genes like *Il10*, *Arg2*, *Tgfb2*, and *Tgfbr3*. In healthy kidneys, RAS-KRM upregulated *Arg2* and *Il10* signaling, important pathways promoting wound healing^38^. Our findings corroborate previous reports that KRM are IL10-producing macrophages in the kidney^19^ and further support their reparative potential (Fig. 4D,G).

RAS induced both pro- and anti-inflammatory genes in all three macrophage populations. RAS-KRM showed higher expression of chemokines (*Ccl2*, *Ccl7*, *Ccl8*, *Ccl20)*, cell adhesion molecules (*Cdh1*, *6*, *11*, *and 16*) and inflammatory genes (*Tnf*, *Il6*, *Nfkb1* and *Il1b*) compared to Sham-KRM. However, CD11c^hi^Mϕ and CD11c^lo^Mϕ showed a greater increase in the expression of pro-inflammatory mediators (*Il1b*, *Ccl2*, *Il6*) compared to RAS-KRM. Interestingly, anti-inflammatory *Il10* and *Arg1* expressed much higher in RAS-KRM, while pro-inflammatory interferon signature genes (*Mx1*, *Irf5*) were downregulated compared to RAS-CD11c^hi^Mϕ and CD11c^lo^Mϕ (Fig. 4E). Thus, monocyte-derived macrophages and KRM upregulated pro- and anti-inflammatory genes, without a clear shift toward either phenotype. Similarly, we observed both pro and anti-fibrotic genes in RAS-KRM. Thus, in ischemic kidneys, KRM activated pro-angiogenic and reparative transcriptional programs, indicating their plastic phenotype.

### In RAS, monocyte-derived KRM fail to upregulate pro-angiogenic and anti-inflammatory genes expressed by native KRM

To compare the gene expression profile of monocyte-derived with native KRM, we flow-sorted KRM from the stenotic kidneys of RAS and RAS + Clodronate mice, and performed qPCR. We observed that expression of pro-angiogenic genes (e.g, *Angpt1*, *Vegfa*) and anti-inflammatory genes (e.g, *Il10*, *Arg1)* was reduced, while the expression of pro-inflammatory genes like *Irf5* and *Nfkb1* increased significantly (Fig. S8,H,I). Thus, monocyte-derived KRM might be occupying KRM niches, but are unable to mimic native KRM function.

### KRM enhance proliferation of peri-tubular endothelial cells

**Figure 5:**
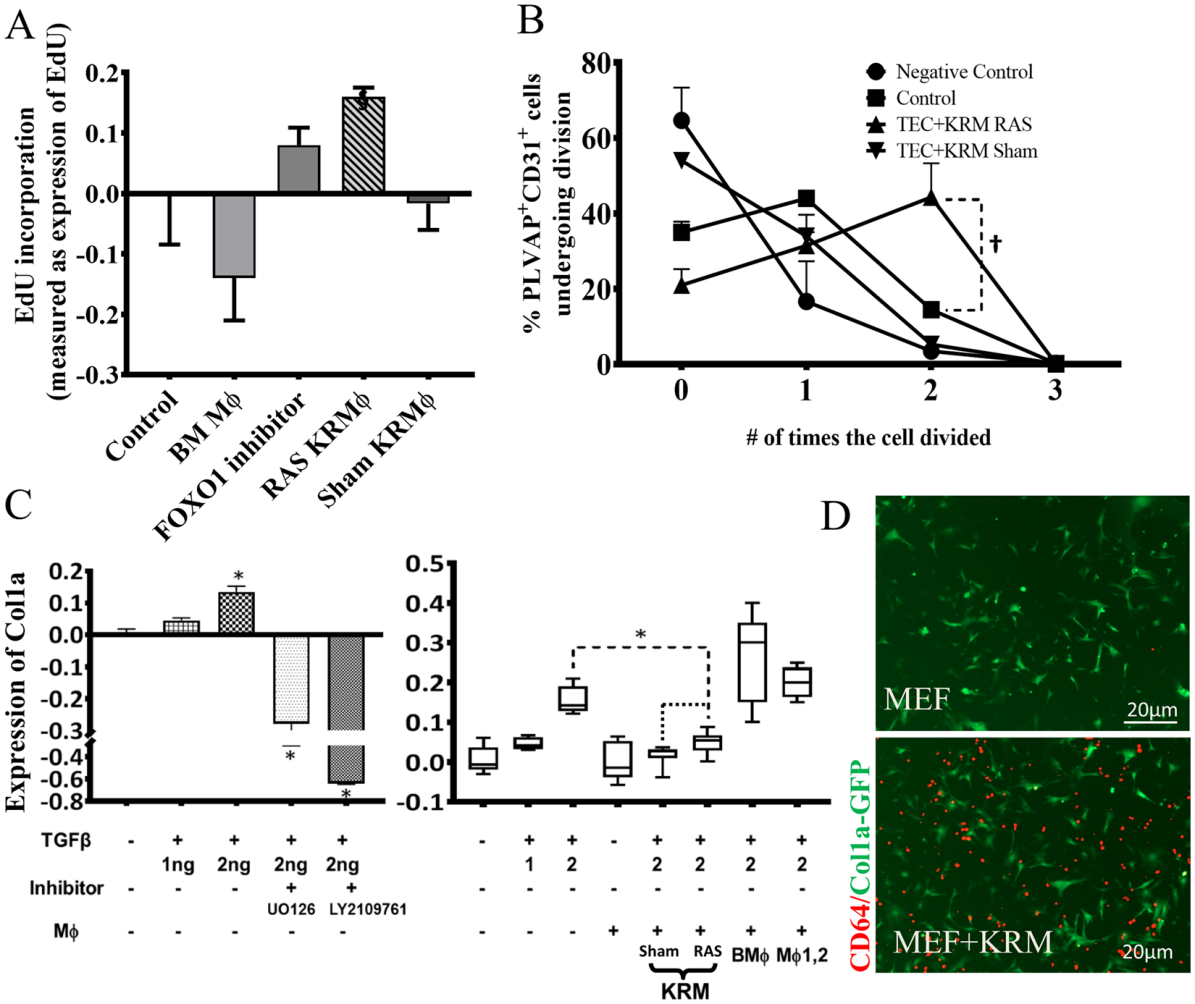
KRM promote proliferation of PLVAP^+^CD31^+^ renal peri-tubular endothelial cells and inhibit TGF-β-induced expression of Col1a *in vitro*. **(A)** Co-culture of RAS-KRM with PLVAP^+^CD31^+^ endothelial cells. EdU incorporation between BM macrophages (Mϕ), RAS-KRM and Sham-KRM compared to FOXO1-inhibitor AS1842856. **(B)** CellTrace Far Red dye dilution assay of PLVAP^+^CD31^+^ cells in a contact co-culture system. **(C)** TGF-β induces dose-dependent expression of Col1a in murine embryonic fibroblasts (MEF) derived from Col1-GFP mice (Lane1–3, Left and right graph). The increase in Col1a was inhibited by UO126 (MEK inhibitor) and LY2109761 (TGF-β receptor I and II dual inhibitors) (Left). Co-incubation of Sham-KRM (Lane5) and RAS-KRM (Lane6) with MEF (GFP) significantly inhibited the increase in Col1a1-GFP (Right), while BMϕ (Lane7) and CD11c^hi/lo^Mϕ (Mϕ1,2) (Lane8) had no effect. BMϕ = bone marrow macrophages; Mϕ1,2 are (n = 5 technical replicates and n = 3 biological replicates per sample); **(D)** Representative images demonstrating contact co-culture of Col1a1-GFP MEFs (green) with (bottom) and without (top) KRM stained with anti-mouse CD64-AF594 (red) *P < 0.01 vs MEF untreated control; ^§^P < 0.01 by One-sample T-test; ^†^P < 0.05 vs Control.

To study the direct contribution of KRM to these pathways, we performed additional *in vitro* experiments. The functional effect of KRM on endothelial cell proliferation was studied by co-incubation with PLVAP^+^CD31^+^ endothelial-cells, which represent renal peritubular capillaries (Figs 2K, S4G). This population was flow-sorted and then co-incubated with either BM-macrophages, RAS-KRM, or Sham-KRM. Proliferation determined by EdU incorporation was greater when co-incubated with RAS-KRM compared to control and Sham KRMs (Fig. 5A). This was further confirmed using dye-dilution experiments, where co-incubation of peri-tubular endothelial cells with RAS-KRM enhanced their proliferation (Fig. 5B).

### KRM inhibit TGF-β-induced Collagen-1α1 expression

RNA-sequencing showed that KRM upregulated gene involved in extracellular matrix remodeling. Therefore, to determine if KRM directly affect fibrosis, we incubated murine embryonic fibroblasts (MEFs), obtained from mice expressing green fluorescent protein (GFP) under the collagen-α1(I) promoter (Col1-GFP) with TGF-β, and measured GFP expression as readout for collagen synthesis. At 18 h, TGF-β induced a dose-dependent increase in GFP signal intensity, reaching statistical significance at 2 ng/ml (Fig. 5C, Left). The dependence on TGF-β signaling was confirmed using UO126 (MEK pathway inhibitor) and LY2109761 (TGF-β receptor inhibitor) (Fig. 5C, Left). Addition of Sham and RAS KRM to MEF co-incubated with TGF-β reduced the GFP signal (Fig. 5C, Right), suggesting that KRM directly counter TGFβ-mediated pro-fibrotic signaling in MEF, possibly related to their ability to upregulate anti-fibrotic genes in renal ischemia^39^. This was not observed in bone-marrow derived macrophages or CD11c^hi/lo^Mϕ.

### CD11c^Int^CD11b^Int^CD68^+^ Macrophages may represent a KRM-like population in human stenotic kidneys

To assess the potential clinical relevance of our findings we initially identified macrophages by flow cytometry in the unaffected portion of a human kidney removed due to renal cell carcinoma. We used a combination of conventional markers like CD68, HLA-DR, CD11b, CD11c, CD14, CD16, and the additional markers CD64 and MerTK^12,40^. Macrophages were classified as Lineage^neg^CD45^+^HLA-DR^+^CD68^+^ and CD11b^+^CD14^+^ but CD16^lo-neg^, indicating a blood-derived origin^5,33,41^. We then further classified macrophages as CD11b^hi^CD11c^hi^, CD11b^int^CD11c^lo-neg^, and a small population of CD11b^hi^ (Fig. S7A). Interestingly, unlike in mice, the expression of CD64 and MerTK was higher in the CD14^hi^CD11b^hi^ subset (Fig. S6B)^40,42^ resembling human dermal CD14^+^ tissue-resident monocyte-derived macrophages that express CD64^42^. CD11b^int^CD11c^lo-neg^Mϕ were CD14^lo^ and thus appear to phenotypically resemble KRM (Fig. S9B).

**Figure 6:**
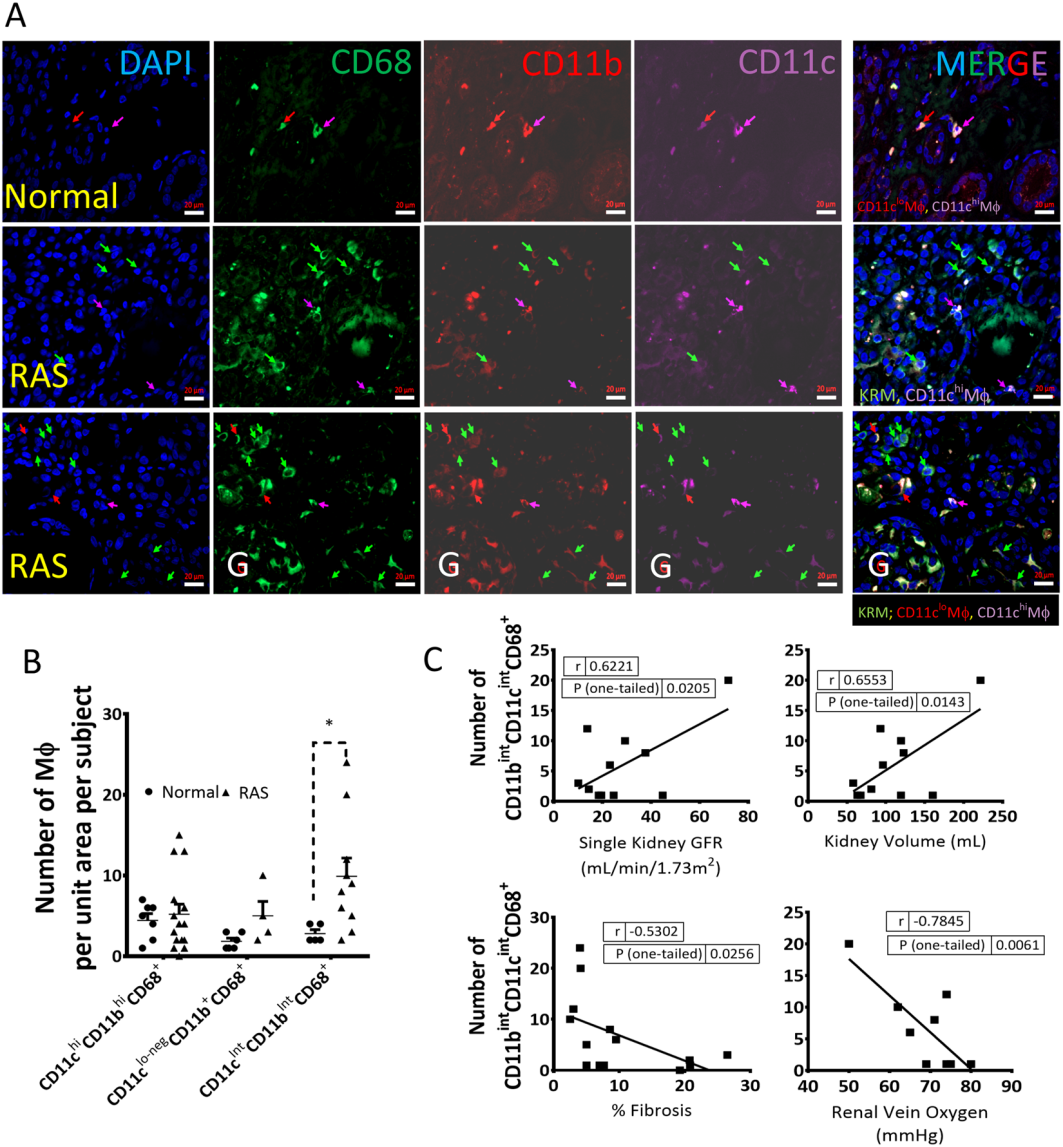
CD11c^Int^CD11b^Int^CD68^+^ macrophage numbers increase in stenotic human kidneys and directly correlate with better kidney function markers. **(A)** Representative images (40 X ) showing DAPI, CD68-AF488 (green), CD11b-AF594 (red), CD11c-AF647 (magenta), and merged (arrows); KRM-like cells were identified as CD11c^Int^CD11b^Int^CD68^+^ in healthy (row 1) and stenotic (rows 2–3) kidneys; G: Glomerulus. **(B)** CD11c^Int^CD11b^Int^CD68^+^ macrophage numbers are significantly higher in RAS compared to normal human kidneys. **(C)** The number of CD11c^Int^CD11b^Int^CD68^+^ correlated directly with GFR and kidney volume, and inversely with renal vein oxygen tension and degree of fibrosis measured by Trichrome staining *P < 0.05 vs Normal (n = 5–7 healthy human kidneys, n = 14 stenotic kidneys).

To determine the effect of RAS on identified human macrophages subsets, we performed immunofluorescence on biopsy samples from healthy kidney donors (time-zero) (n = 7) and stenotic kidneys of RAS patients (n = 14) included in an on-going study (Table 2S1)^5^. In healthy normal and RAS kidneys, ~50% of CD68^+^ cells were also CD64^+^, whereas <20% expressed both CD64 and MerTK (Fig. S7B), indicating heterogeneous expression. Flow cytometry results were replicated using immunofluorescence studies, as we identified similar populations of CD11c^Int^CD11b^Int^CD68^+^, CD11c^lnt^CD11b^+^CD68^+^, and CD11c^hi^CD11b^hi^CD68^+^ macrophages (Fig. 6A).

The number of KRM-like CD11c^Int^CD11b^Int^CD68^+^ increased in stenotic compared to healthy human kidneys (Fig. 6B), correlated directly with stenotic-kidney GFR and volume, and inversely with renal vein oxygen level and fibrosis (Fig. 6C). Hence, KRM-like CD11c^Int^CD11b^Int^CD68 + Mϕ may play a reparative role in the human kidney.

## Discussion

This study shows that F4/80^bright^CD64^+^CD11c/CD11b^int^ are murine KRM with reparative potential. KRM self-renewed in stenotic kidneys. Irradiation and clodronate-induced depletion of KRM led to their replenishment via donor-derived bone marrow monocytes, but in superimposed RAS, native KRM depletion amplified the loss of peritubular capillaries, suggesting functional deficit of monocyte-derived KRM compared to native KRM. Indeed, native KRM in RAS transcriptionally upregulated expression of pro-angiogenic and wound healing pathways, capable of initiating a reparative transcriptional program to limit kidney damage. *In-vitro*, RAS-KRM promoted peritubular endothelial cell proliferation and blunted TGF-β-induced collagen-1 production. Furthermore, KRM homologues expand in human stenotic kidneys, and correlate with better function.

Our previous studies in humans and swine have shown that RAS leads to irreversible microvascular rarefaction^43–45^. Our murine model of RAS mimics the human RAS and demonstrates renal ischemia, hypertension, reduced glomerular function^46^ and fibrosis^47^ that in turn leads to renal inflammation and loss of peritubular capillaries and thus is a relevant model for chronic sterile renal injury. Hence, we have observed association of macrophages with RAS in mice, swine, as well as human RAS kidneys.

Renal macrophages have divergent phenotypes, gene expression profiles, and responses to physiologic stimuli^11,19^, which we linked to their origin. Using fate-mapping and parabiosis studies, we identified KRM, classical monocyte-derived CD11c^lo^Mϕ, and non-classical monocyte-derived CD11c^hi^Mϕ. Among these, KRM were the most abundant in the healthy mouse kidney, self-maintained, and progressively self-renewed in the stenotic kidney, whereas CD11c^hi^Mϕ and CD11c^lo^Mϕ were circulation-derived and short-lived. Consistent with previous studies^10,22^, quiescent KRM expressed typical markers of TRM. Thus, our data are congruent with the notion that resident-macrophages are long-lived and capable of self-maintaining^10,21,22,48^.

Irradiation depleted native KRM and unsealed tissue niches that were then occupied by monocytes from donor bone marrow thus giving rise to monocyte-derived KRM^29^. However, while phenotypically comparable to KRM, a superimposed ischemic stress revealed that those cells could not be sustained, and a decline in their numbers in RAS was associated with capillary loss. Continuous administration of low-dose clodronate had a similar effect. Importantly, we observed that monocyte-derived KRM formed in the progressive RAS had greater expression of pro-inflammatory genes than native KRM. Similarly, in injured hearts monocyte-derived macrophages have limited ability to restore TRM, and when restored TRM lack the reparative function of their embryonically-derived counterparts^49^. BM-derived Kupffer cells, despite normal density and location, also have reduced phagocytic activity^31^. Similar phenomenon has been recently demonstrated in lung^35^. Monocyte-derived alveolar macrophages proved to be profibrotic compared to native, alveolar TRM. Thus, although KRM ostensibly repopulate after BMT, the new subset resembles their native counterparts phenotypically by surface markers, rather than functionally. Contrarily, irradiation might have been adversely affected the niche by inducing cell cycle arrest and senescence. Future studies are needed to define whether a longer homing time would allow new cells to acquire the functional attributes of bone-fide KRM^29^.

Our transcriptional profiling agrees with the earlier report that at steady state *Fcgr4* is upregulated in KRM compared to CD11c^hi^Mϕ and CD11c^lo^Mϕ^11^. Similarly, KRM differentially expressed microglia marker *Tmem119* but not the Kupffer cell marker *Clec4f* ^48,50^. More importantly, we identified *Spp1*, *Mmp12* and *Mmp13* as genes that are not expressed in other TRM or other kidney cells, but unique to KRM. We validated *Mmp12* and *Mmp13* transcripts in kidney macrophages using the mouse cell atlas^51^ and kidney single-cell atlas^52^; however, *Spp1* was present in many other kidney cells. Interestingly, while *Mmp12* and *Mmp13* expression decreased in RAS-KRM, *Spp1* increased. Further studies will highlight the role of these genes in the kidney diseases. Unlike Kupffer cells or Microglia, RAS-KRM show genes that are differentially expressed in functional units of kidney such as thick-ascending limb (*Umod*, *Slc12a1* and *Cldn10*)^53^, intercalated cells of collecting duct (*Aqp6*, *Idh3a*, *Slc4a1*)^53^, distal convoluted tubule (*Slc12a3*, *Tmem52b*, *Atp1a1*), parietal cells (*Aqp2*) and podocytes (*Bcam*)^52^. Tissue-resident macrophages at steady state are not known to express these genes, therefore we speculate that in ischemic conditions, KRM may efferocytose apoptotic or senescent kidney cells, or phagocytose the extracellular vesicles released by the damaged cells thereby expressing their genes. Phagocytosing EVs may be unique to KRM because they form an anatomical and functional unit with endothelial cells that monitors the transport of small particles^11^. Further studies are needed to identify genes or transcription factors that promote efferocytosis in KRM.

In RAS kidneys, the overall increase in all macrophage populations was intensified with increased fibrosis and loss of peri-tubular endothelial cells, but only KRM responded by upregulating pro-angiogenic and wound-healing pathways, which are vital for repair by supplying the newly formed tissue with nutrients and oxygen. Top 100 DEGs in the ischemic kidney KRM include the Vegf family of genes that initiate the vessel sprouting. TRM initiate VEGF-dependent vascular anastomosis to form vascular networks^54^. PLVAP, a modulator of VEGF-induced angiogenesis^55^, contributes to peritubular capillary formation^56^. Our studies indicate that *in vivo*, depletion of native KRM using clodronate was associated with loss was peri-vascular endothelial cells. In culture KRM promoted proliferation of PLVAP^+^CD31^+^ peritubular endothelial cells and attenuated an increase in TGFβ*-*induced *Col1a1* expression. The ability of RAS-KRM to promote endothelial cell proliferation and attenuate collagen formation supports the notion that KRM may possess reparative properties. Indeed, resident-macrophages may facilitate resolution of fibrosis in kidney^32,57^ and liver injury^57^, possibly via formation of functional physiological units with endothelial cells^11,58^. In BMT + RAS, loss of KRM may have disrupted these units, magnifying loss of peri-tubular capillaries.

We identified human homologues of KRM as CD11b^int^CD11c^int^CD68^+^. Renal CD11c^hi^ are considered as DC, and CD11b^hi^CD11c^hi^CD68^+^ may be either macrophages or DCs^33,59^. Elevated CD11b^int^CD11c^int^CD68^+^ macrophages in stenotic human kidneys align with our observations in mice. Importantly, their numbers directly correlated with better kidney function and oxygenation, and inversely with fibrosis and atrophy, providing potential clinical support for their functionally consequential reparative role.

Our study may bear possible limitations. Despite a relatively brief period of RAS, stenotic murine kidneys recapitulate many pathological events seen in human and larger animal RAS^60^. Besides, macrophages, smaller populations of monocytes and DCs likely also contribute in RAS. We have studied transcriptomics at 4 weeks of RAS, which may represent tissue repair/reorganization phase; studying earlier time-points may help identify pro-fibrotic macrophage subsets. Stenotic-kidney KRM appear to heterogeneously express both pro- and anti-inflammatory and -fibrotic genes, but a KRM sub-population might have possibly dictated some of these expression patterns. Single-cell RNA-sequencing studies may help elucidate this possibility. We could not fully define the extent to which reciprocal changes in inflammatory macrophages vs. KRM regulate kidney damage. While low-dose clodronate selectively deleted KRM amplifying fibrosis, we cannot rule out neutrophil contribution to this effect. Quantification of KRM in human kidney could have thresholding artifacts, which may contribute to some discrepancy between flow cytometry analysis and immunofluorescence.

In summary, our results suggest that KRM are a unique subset of renal macrophages, phenotypically equivalent to fate-mapped renal CD11b^int^F4/80^hi^ macrophages that are tissue-resident, self-maintain locally, and replenish slowly^22^. In response to microvascular rarefaction in stenotic mouse kidneys, KRM transcriptionally upregulate proangiogenic and wound healing pathways bearing a potential to repair damaged tissues. Human homologues of KRM identified as CD11b^int^CD11c^int^CD68^+^ increase in stenotic kidneys and correlate with kidney vitality. Further studies to exploit KRM, may open therapeutic avenues for treatment of chronic renal disease.

## Methods

The study protocol was approved by Mayo Clinic’s Institutional Animal Care and Use Committee (Protocol numbers A00001844-16 and A32415-16) and all experiments were performed in accordance with IACUC guidelines and regulations. Most RAS procedures were performed on n = 30–50; 20-week-old wild-type C57BL/6 J mice (Jackson Laboratories). Syngeneic mice expressing CD45.1 and wild type CD45.2 were used for Parabiosis and BMT studies.

### Induction of Renal artery stenosis (RAS)

To induce RAS, mice were anesthetized with 1.75% isoflurane supplemented with O_2_ and placed prone on a heating pad at 37 °C. The right kidney was exposed by a flank incision and its renal artery bluntly dissected from the renal vein. A 0.15 mm (ID × 0.5 mm) long polytetrafluoroethylene tube (Braintree Scientific, Braintree, MA) cuff was placed around the right renal artery and tied with 10–0 nylon sutures. Kidneys were then returned to their original positions and the incisions sutured. Blood pressure was measured before RAS or Sham surgery (baseline) and at 2, 4, and 6 weeks after surgery by tail-cuff, using an XBP1000 system (Kent Scientific, Torrington, CT). Sham surgery consisted of isolation of the renal artery without placement of a cuff ^61^. At day 3, 7, and 28 days post-RAS 4–6 mice were euthanized to assess expansion of macrophages.

### Fate-mapping studies and Tamoxifen dosing

B6.129P2(C)-Cx3cr1tm2.1(cre/ERT2)Jung/J (Jackson Labs #020940) were crossed to B6.Cg-Gt(ROSA)26Sortm14(CAG-tdTomato)Hze/J (Jackson Labs #007914). The F1 offspring were Cx3cr1^CreER+/−^Rosa26^+/−^. At 6–8 weeks of age mice of both sexes were injected intraperitoneally tamoxifen (Sigma) prepared in warm ethanol and mixed with corn oil. Around 75 mg tamoxifen/kg body weight was injected intraperitoneally to Cx3cr1^CreER+/−^Rosa26^+/−^ induce recombination for 5 consecutive days. Mice were euthanized at 4 weeks and only the resident-macrophages were tdTomato positive. At 20 weeks of age, RAS/Sham surgeries were performed on n = 20 (n = 10 per group) tamoxifen injected Cx3cr1^CreER+/−^Rosa26^+/−^ mice^22^.

### Parabiosis

6–8-week-old C57BL/6 (n = 4) congenic CD45.1 and CD45.2 mice were surgically connected in parabiosis as previously described^20,62,63^. After corresponding lateral skin incisions were made from elbow to knee in each mouse, forelimbs and hindlimbs were tied together using suture and the skin incisions were closed using stainless steel wound clips (Fine Scientific Tools Inc, USA). After surgery, mice were maintained on a diet supplemented with trimethoprim/sulfamethoxazole for prophylaxis of infection. 10 weeks after the parabiosis surgery the mice were euthanized, perfused and kidneys were harvested. Detail methods for tissue digestion, single cell preparation, flow cytometry, RNA-sequencing and validation by gene expression are provided in Supplemental Methods.

### Patient Protocol

Patients were identified as part of a clinical investigation of tissue oxygenation in human renovascular disease between 2008 and 2012. Informed, written consent was obtained after receiving approval from the Mayo Clinic’s Institutional Review Board in adherence with the Declaration of Helsinki. A 3-day inpatient protocol was performed in the Clinical Research Unit of St. Mary’s Hospital, Rochester, Minnesota. Fourteen patients underwent transvenous biopsy of the right-sided stenotic kidney via the jugular vein. Inclusion criteria were the presence of unilateral right-sided ARAS >70% obstruction, as previously described^64^ (Table S1).

For the healthy group, Implantation biopsies obtained from 15 living kidney donors, selected to have a similar distribution of age and sex, were identified from the Mayo Kidney transplant program as previously described^65^. All research was performed in accordance with Mayo Clinic’s IRB regulations. Detailed methods and hemodynamic data for RAS patients is elaborated in Supp Methods.

### Immunofluorescence labeling of human kidney biopsies

Non-tumor pieces of kidneys were obtained from patients undergoing nephrectomy for renal cell carcinoma. Informed, written consent was obtained after receiving approval from the Mayo Clinic’s Institutional Review Board (IRB#16–009485) in adherence with the Declaration of Helsinki. All research was performed in accordance with Mayo Clinic’s IRB regulations. These kidney pieces were enzymatically digested and subjected to flow cytometry to identify macrophage markers (Table S3). Informed, written consent was obtained after receiving approval from the Institutional Review Board of the Mayo Clinic in adherence with the Declaration of Helsinki from all patients.

### Statistics

All statistics and graphs were generated using GraphPad Prism 7.1, and data presented as Mean ± S.E.M. One-sample t-test was used for comparing resolution metric (Rd). For gene expression and percent macrophages of total cells, unpaired Student t-test or Mann-Whitney test was applied, and P < 0.05 considered significant. Multiple groups were tested for significance using ANOVA followed by Dunnett’s multiple comparisons test. For RNAseq, pairwise comparisons between macrophage populations (CD11c^hi^Mϕ, CD11c^lo^Mϕ, and KRM), as well as comparisons between sham and RAS for each macrophage population, were conducted by applying Wald test of the negative binomial distribution to the log2 gene counts using the DESeq. 2 statistical package^66^, and genes that showed statistically significant differences were selected (fold-change > 2, P < 0.05).

### Accession numbers

The accession number for the RNAseq data reported in this paper is NCBI GEO: GSE116094.

## Electronic supplementary material

Supplementary Figures Tables and Methods

Supplementary Information 2

Supplementary Information 3
