# Supplementary material for: Kidney-resident macrophages promote a proangiogenic environment in the normal and chronically ischemic mouse kidney
Source: Sci Rep. 2018 Sep 17;8:13948. doi: 10.1038/s41598-018-31887-4 (PMC6141464; doi:10.1038/s41598-018-31887-4)
Supplement: Supplementary file 1 — Supplementary Figures Tables and Methods [file 41598_2018_31887_MOESM1_ESM.pdf]

Kidney-resident macrophages promote a proangiogenic environment in the normal and chronically ischemic mouse kidney

Amrutesh S. Puranik[1,11] Ph.D., Irina Leaf[9] Ph.D., Mark A. Jensen[2] Ph.D., Ahmad F. Hedayat[1] M.D., Ahmad Saad[1] M.D., Ki-Wook Kim Ph.D[10]., Abdulrahman M. Saadalla[4] M.D., John R. Woollard[1], Sonu Kashyap[3] Ph.D., Stephen C. Textor[2] M.D., Joseph P. Grande[3] M.D., Ph.D., Amir Lerman[5] M.D., Robert D. Simari[6] M.D., Gwendalyn J. Randolph[10], Ph.D., Jeremy S. Duffield[7, 8] M.D., Ph.D., Lilach O. Lerman[1*] M.D., PhD

Supplementary Figure 01:

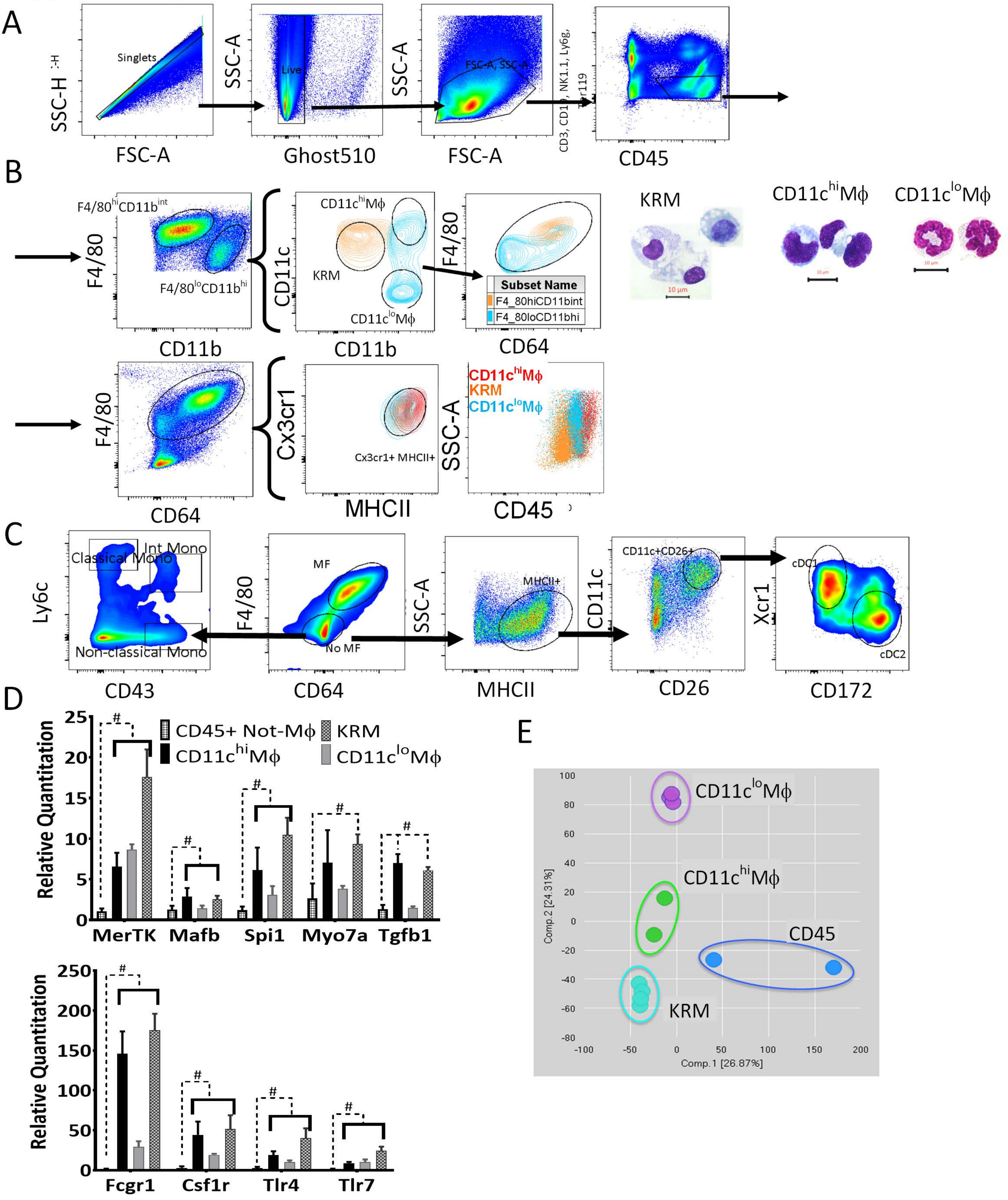

Figure S1: Renal macrophages comprise of transcriptionally unique long-lived kidney-resident macrophages and monocyte-derived CD11c^{hi} and CD11c^{lo} macrophages.

(A) Cells from Sham kidneys of C57BL/6 mice were prepared as described in Materials and Methods. Singlets (FSC-H vs. FSC-A), Ghost510^{neg} Live, Lineage^{neg} kidney lymphocytes gated as FSC-A SSC-A followed by Lineage^{neg}CD45^{+} were gated on CD64$^{+/lo}$F4/80^{+} in Figure 1. The Lineage-positive cells marked by Ly6G, CD3, CD19, NK1.1 were gated out. **(B – top row)** Macrophages were gated using F4/80 vs. CD11b. Kidney-resident macrophages (KRM) were gated as F4/80^{hi}CD11b^{int} and monocyte-derived macrophages as F4/80^{lo}CD11b^{hi}. For validating our gating strategy, both these populations were overlayed on on CD11c vs CD11b. The F4/80^{hi}CD11b^{int} were the CD11b^{int}CD11c^{int} and therefore KRM while F4/80^{lo}CD11b^{hi} were CD11c^{lo}Mf and CD11c^{hi}Mf. Furthermore, overlay of these populaitons on F4/80 vs CD64 plot show that the CD11c^{hi}Mf and CD11c^{lo}Mf are F4/80^{lo}. Analysis of cytospin preparations of flow sorted subpopulations after Wright -Giemsa staining and observed under 100X showed that KRM had typical structural features of tissue macrophages, being large with abundant foamy cytoplasm and prominent cytoplasmic vacuoles. **(B - bottom row)** Overlay of CD11c^{hi}Mf (red), CD11c^{lo}Mf (blue) and KRM (orange) gated on Cx3cr1 vs MHCII and SSA vs CD45. **(C)** Monocytes were classified from non-macrophages population using Ly6c vs CD43. Classical monocytes were defined as Ly6c^{+}CD43^{lo-neg}, Non-classical monocytes were Ly6c^{neg}CD43^{+}, intermediate monocytes were Ly6c^{int}CD43^{int}. The non-macrophages were also gated for MHCII^{+} dendritic cell (DCs) subsets: MHCII^{+}CD11c^{hi}CD26^{+}XCR1^{hi} as cDC1 and MHCII^{+}CD11c^{hi}CD26^{+}CD172^{hi} as cDC2. **(D)** Taqman Low Density Array (TLDA) of KRM, CD11c^{hi}Mf, CD11c^{lo}Mf compared to not-Mf CD45^{+}. Expression of selected genes in CD11c^{hi}Mf, CD11c^{lo}Mf and KRM. **(E)** Principal component analysis (PCA) showed the strongest separation between not-MfCD45^{+} and other macrophage populations accounting for 26.87% of variance in the first principal component. Separation between CD11c^{hi}Mf, CD11c^{lo}Mf and KRM was observed in the second principal component with variance 24.31%. The transcriptome of CD11c^{lo} appeared to be distinct from CD11c^{hi} and KRM while CD11c^{hi} and KRM were located closer in the PCA plot and therefore, were expected to have more similar transcriptional profiles. KRM n=4, CD11c^{lo}Mf n=3 and CD11c^{hi}Mf n=2. A-D and F. Data represent n=4 independent experiments with at least n=3 mice per group. Data is represented as mean±S.E.M. #P<0.01 vs CD45^{+}Mf

Supplementary Figure 02:

F4/80^{Bright} CD64^{+}Kidney-resident macrophages (KRM)

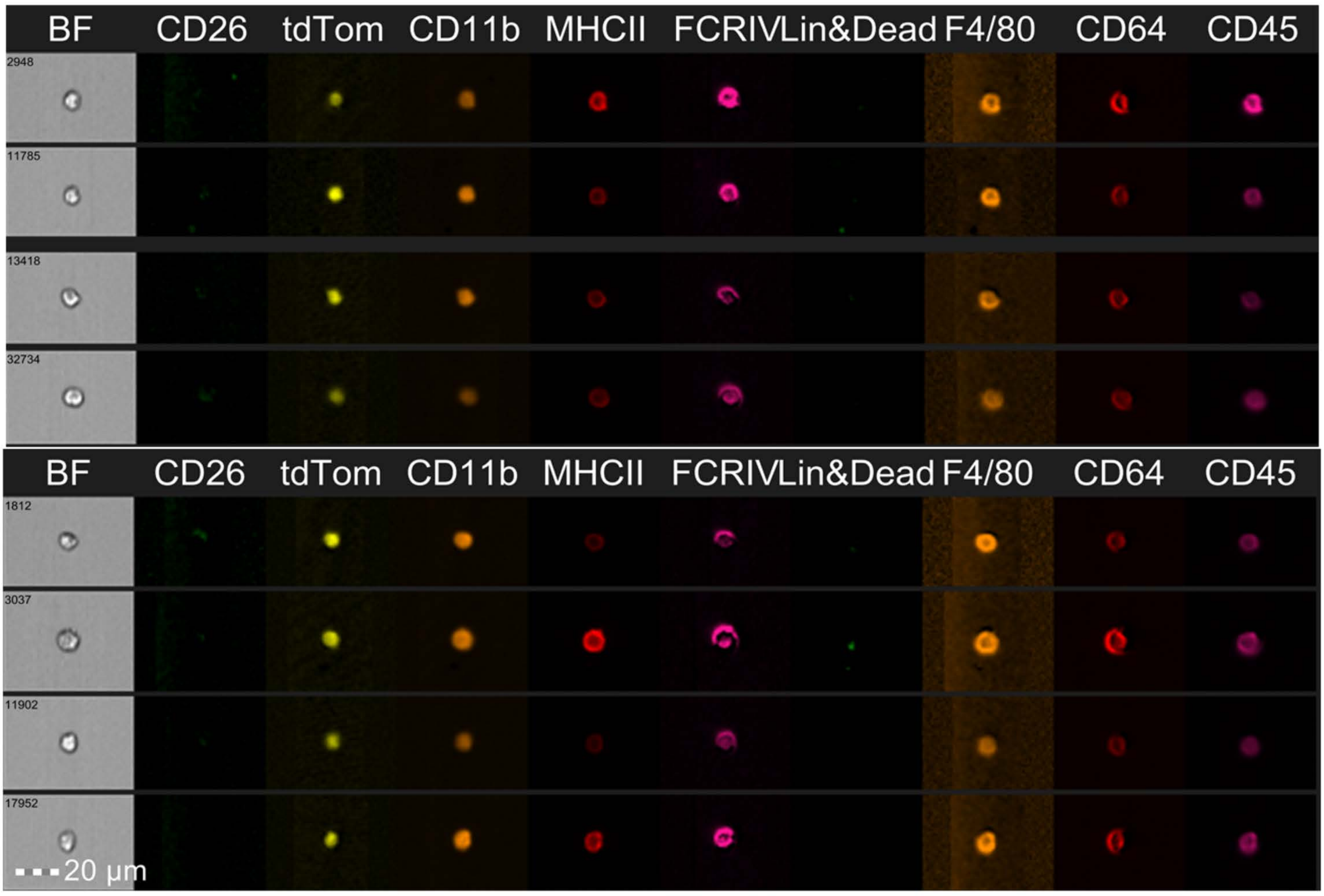

F4/80^{int} CD64$^{lo\text{-}neg}$CD11b^{hi} Monocyte-derived macrophages

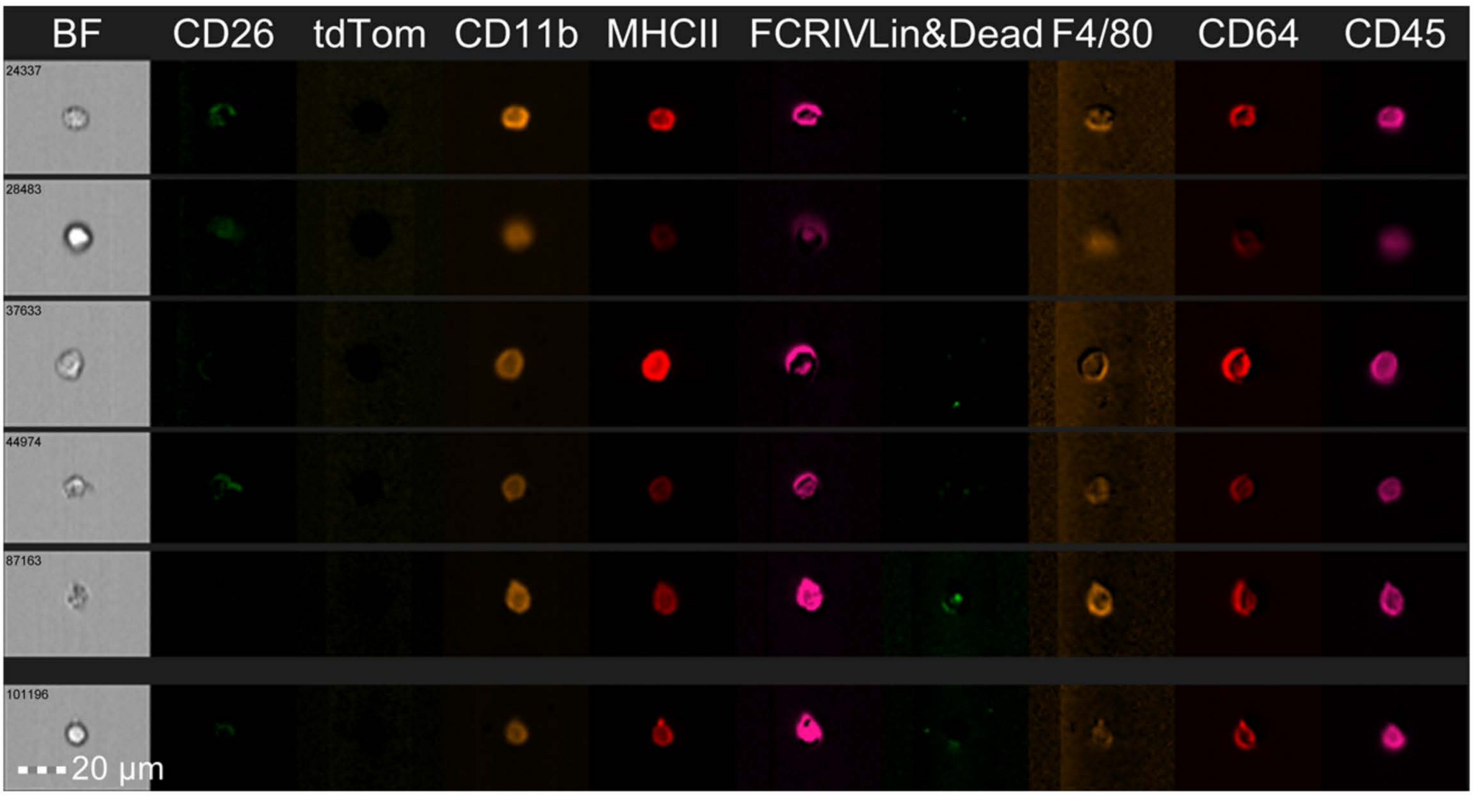

Figure S2: Validating the Gating Strategy by Imaging cytometry: F4/80^{Bright} and F4/80^{int} macrophages are FCRIV^{+}

The gating strategy was validated by studying F4/80^{Bright} and F4/80^{int} macrophages using FlowSight^{TM}. The F4/80^{Bright} population was CD64^{+}tdTomato^{+}CD11b^{int}CD11c^{int} and CD45^{lo}, representing kidney-resident macrophages, while F4/80^{int} population was CD64^{dim}tdTomato^{-}CD11b^{hi}CD11c^{+} and CD45^{hi} macrophages, representing CD11c^{lo}Mϕ or CD11c^{hi}Mϕ. Both F4/80$^{Bright\ and\ int}$ populations were FCRIV^{+}.

Supplementary Figure 3:

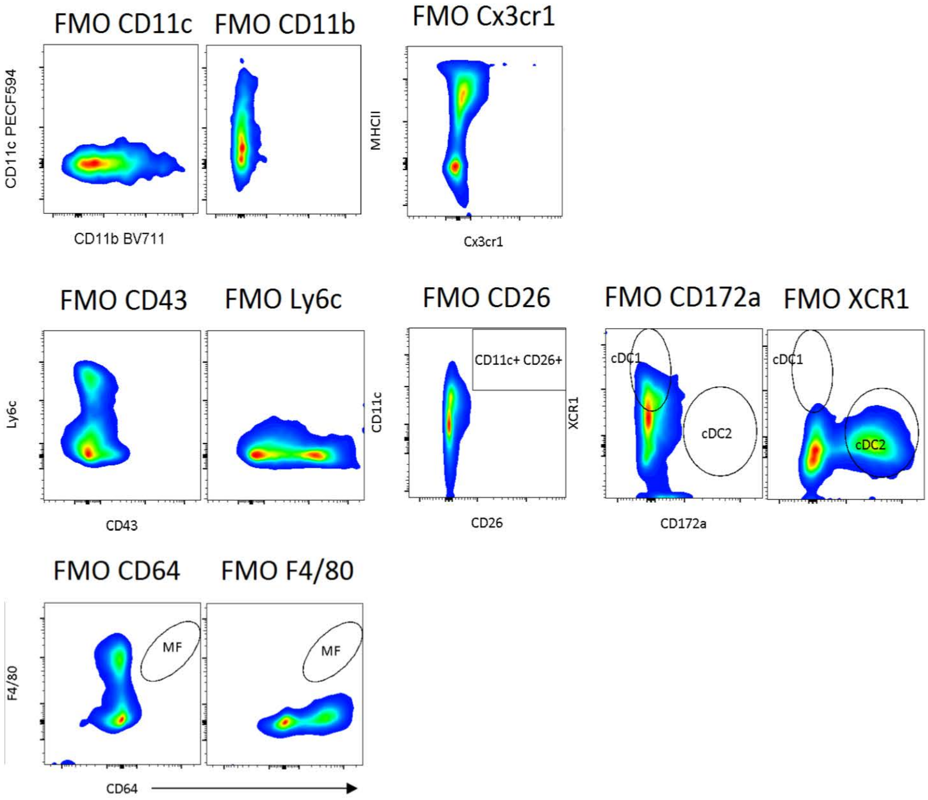

Figure S3: Fluorescence minus one (FMO) controls
FMO controls used for CD11b, CD11c, Cx3cr1, CD43, Ly6c, CD26, Cd172a, Xcr1, CD64 and F4/80, used for gating in Figure 1.

Supplementary Figure 04

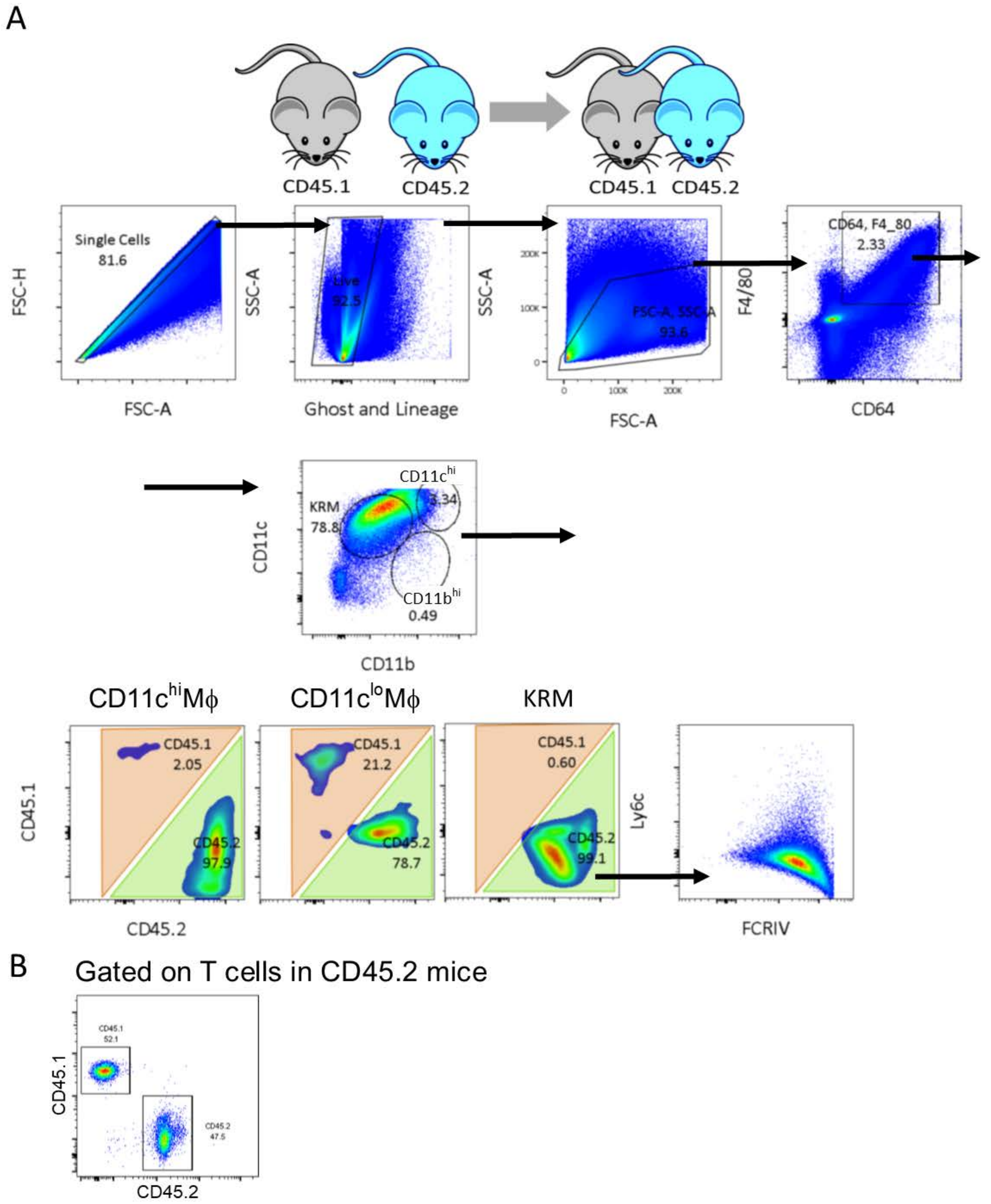

Figure S4: Parabiosis Studies

(A) C57BL/6 congenic CD45.1 and CD45.2 mice (n=4 each) were surgically connected in parabiosis. Macrophages in the dissociated kidney were identified as Singlets (FSC-H vs. FSC-A), Ghost510^{neg} Live, Lineage^{neg} kidney lymphocytes gated as FSC-A SSC-A followed by CD64^{+}F4/80^{+}. These macrophages were further gated in to three populations based on the expression of CD11b and CD11c, as described. KRM demonstrated the smallest contribution (<1%) in CD45.2 from the parabiont partner CD45.1. CD11c^{hi}Mf showed around 2%, while CD11c^{lo}Mf, a small population, showed around 15-20%. **(B)** Representative image of T-cells in the blood of CD45.2 mice. This data showed around 47% chimerism between parabionts, n=4 mice. Mouse images adopted from https://openclipart.org/detail/17558/simple-cartoon-mouse.

Supplementary Figure 5:

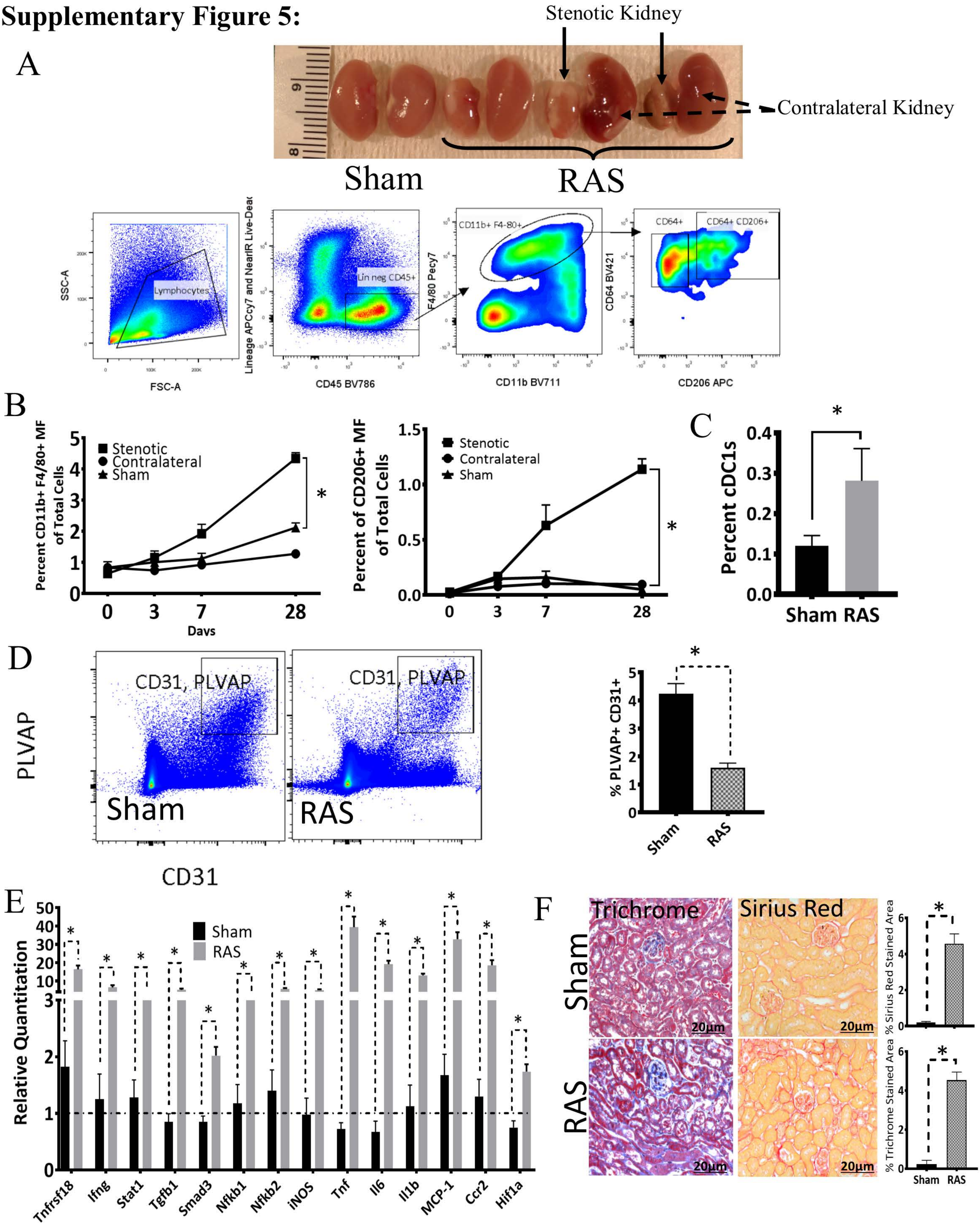

Figure S5: Effect of Renal artery stenosis on ischemic kidney

(A) Representative photos of the Sham, stenotic and contralateral kidneys with Scale depicting smaller stenotic and enlarged contralateral kidney in RAS mice compared to Sham. Gating strategy for flow cytometric identification of total macrophages. We identified macrophages as lineage and live-dead negative $CD45^{+}CD11b^{+}F4/80^{+}CD64^{+}$. Mannose receptor positive (CD206+) macrophages were identified as lineage and live-dead negative $CD45^{+}CD11b^{+}F4/80^{+}CD64^{+}CD206^{+}$. **(B)** The total number of macrophages and $CD206^{+}$macrophages in stenotic and contralateral kidneys of RAS and Sham mice on days 0, 3, 7, and 28; n>4 per time point, **(C)** The percent of cDC1 in RAS kidneys increased compared to Sham. **(D)** Flow cytometry to identify PLVAP and CD31 (pan-endothelial antigen) in Sham and RAS Kidneys. RAS reduced $PLVAP^{+}CD31^{+}$ cells. **(E)** Pro-inflammatory gene expression increases significantly in whole RAS kidney as compared to Sham. **(F)** Trichrome and Picro Sirius Red Staining for Sham and RAS with quantification. Data is represented as n=6 mice mean±S.E.M. *P < 0.01 vs Sham. Kidney images in **5A** captured by a handheld phone camera.

Supplementary Figure 6:

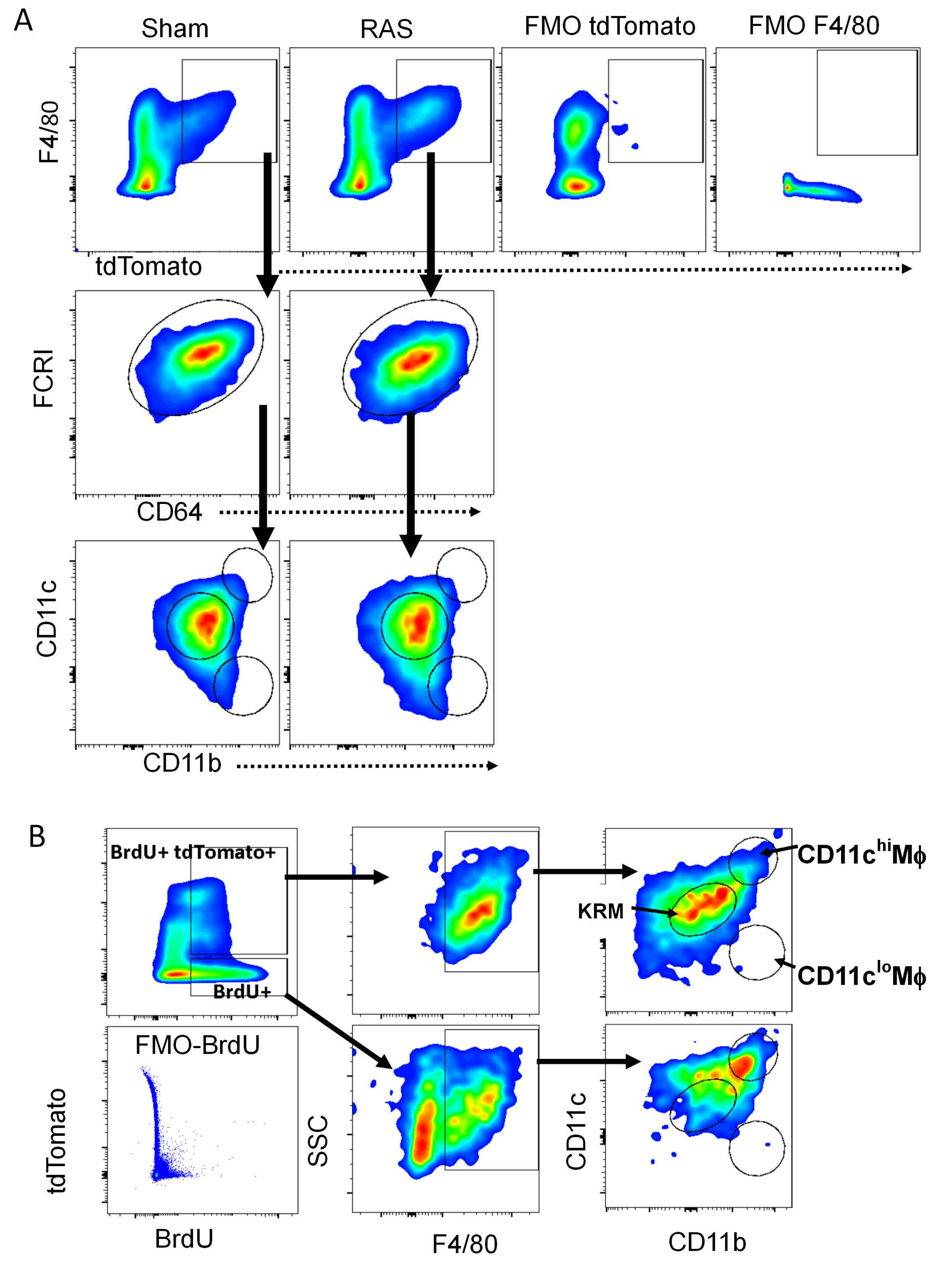

Figure S6: Gating Streategy to identify BrdU^{+} KRM in CX3CR1^{creER}:Rosa26-tdTomato reporter mice
(A) Fate-mapping studies using tamoxifen treated CX3CR1^{creER}:Rosa26-tdTomato reporter mice show that F4/80^{+}tdTomato^{+} gated macrophages were FCRIV^{+}CD64^{+} in Sham and RAS. Furthermore, these cells were CD11b/c^{int}, and therefore KRM. FMO-F4/80 and FMO-tdTomato were used to for gating F4/80^{+}tdTomato^{+}. **(B)** Gating strategy for measuring BrdU-positive population. Live, Lineage^{neg}CD45^{+} population gated as tdTomato vs BrdU. tdTomato^{+}BrdU^{+} population gated as F4/80^{+}macrophages. Further, this population gated as CD11b vs CD11c to identify CD11c^{hi}Mϕ, CD11c^{lo}Mϕ and KRM demonstrating the majority of proliferating tdTomato^{+} population were KRM. tdTomato^{-}BrdU^{+} gated as macrophages were CD11c^{hi}Mϕ (n=6).

Supplementary Figure 7:

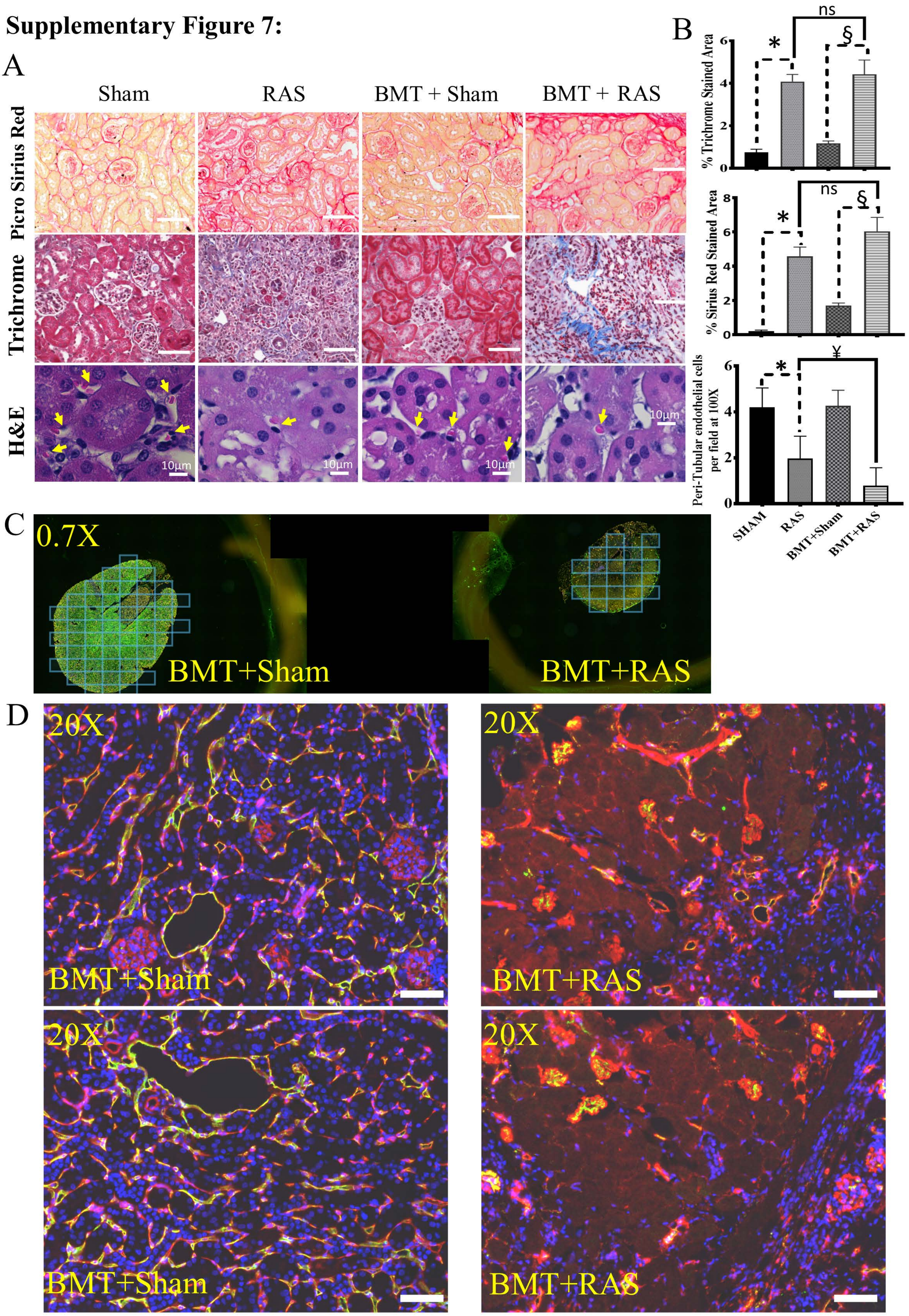

Figure S7. Loss of PLVAP+CD31+ cells is amplified in BMT+RAS.

(A, B) Trichrome and Picro-Sirius Red stains do not show more fibrosis in BMT+RAS kidneys than in RAS (40X). Hematoxylin and Eosin (H&E) images (100X) of BMT+RAS show a fall in the numbers of peritubular capillary endothelial cells (yellow arrows). RAS shows tubular atrophy. n=6 mice/group; *$P \leq 0.01$ vs Sham; §$P<0.05$ vs BMT+Sham; ¥$P<0.01$ vs RAS. **(C)** Representative Formalin fixed paraffin embedded (FFPE) section of BMT+Sham (Left) BMT+RAS (Right) stained for PLVAP+CD31+ imaged using Vectra at 0.7X. The square regions drawn are the area imaged at 20X. Percent of PLVAP+CD31+ cells are counted from the square regions and averaged per section per group. **(D)** Representative image of BMT+Sham (Left) and BMT+RAS (right) showing PLVAP (green) CD31 (red) staining. It is observed that while CD31 stains glomerular cells and peri-tubular cells, PLVAP preferentially stains peri-tubular cells. In BMT+RAS the number of PLVAP+CD31+ cells are reduced.

Supplementary Figure 8:

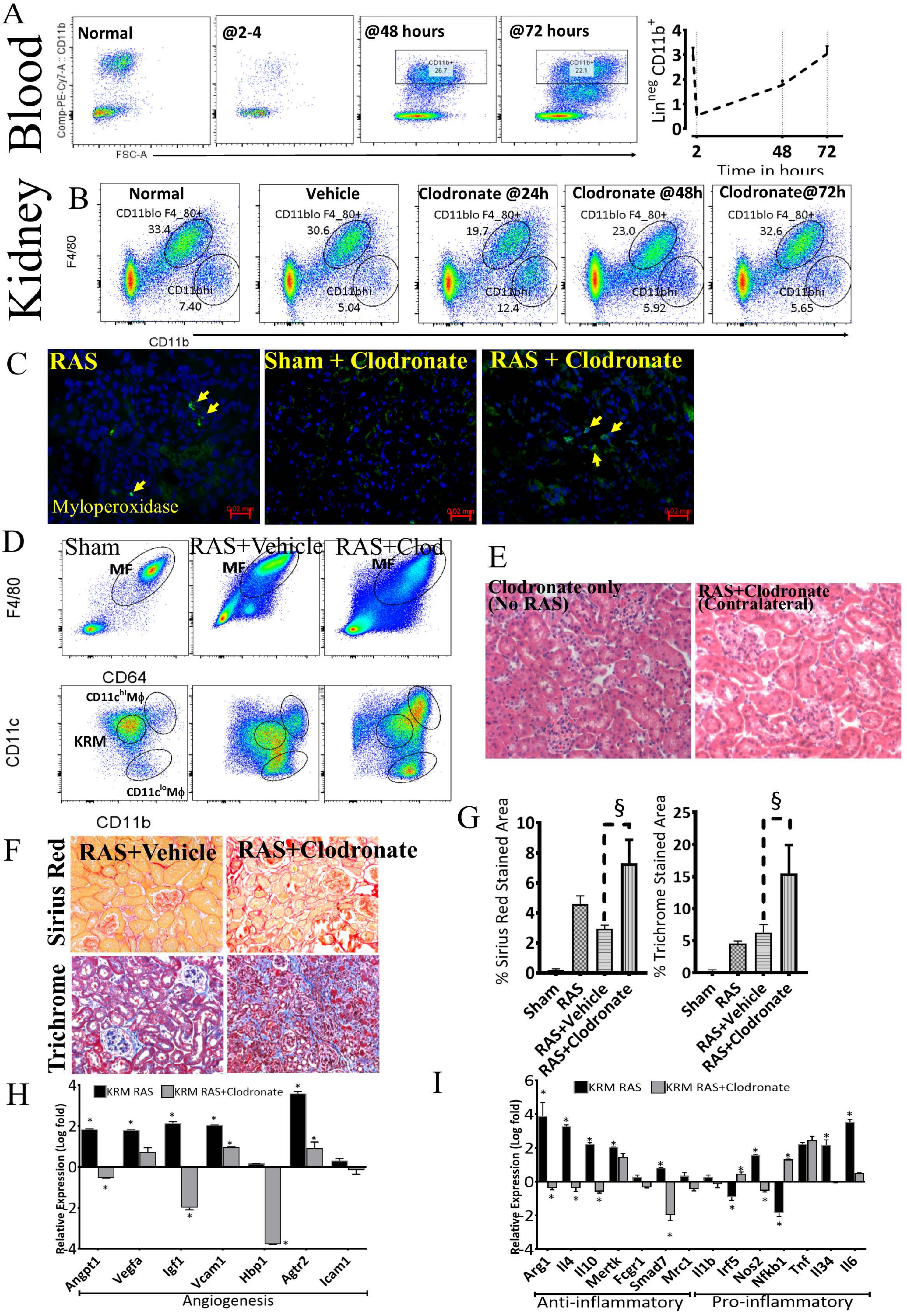

Figure S8. Effect of Liposomal Clodronate on blood monocytes and Normal, Stenotic and Contralateral Kidneys. (A) At single dose liposomal clodronate reduces blood monocytes, which are replenish by 72 hours (graph). **(B)** Flow cytometry of a Lineage and Ghost510^{neg}CD45^{+} lymphocytes, followed by CD11bVsF4/80. In the normal kidney, at single dose, clodronate reduced (but failed to abolish) the KRM, that are replenished in 72 hours. **(C)** Immunofluorescence Staining for Myloperoxidase in RAS, RAS+Clodronate and Sham+Clodronate. Administration of low-dose clodronate does not increase myeloperoxidase (MPO). **(D)** Flow charts of gating strategy (macrophages identified as F4/80+CD64+ followed by CD11b vs CD11c) demonstrating that administration of low-dose clodronate for 4 weeks significantly depletes KRM. **(E)** Chronic administration of low-dose clodronate has no effect of kidney tubules as depicted by H&E staining. **(F)** Stenotic kidney representative images showing trichrome and Sirius red in RAS+Vehicle and RAS+Clodronate.**(G)** Quantitation of Trichrome and Sirius red staining area, in Sham, RAS, RAS+Vehicle and RAS+Clodronate groups n=6, §P<0.01.**(H, G)** Gene expression in RAS-KRM (native-KRM) and RAS+Clodronate KRM (monocyte-derived KRM) (n=4) P<0.05 Vs Sham.

Supplementary Figure 9.

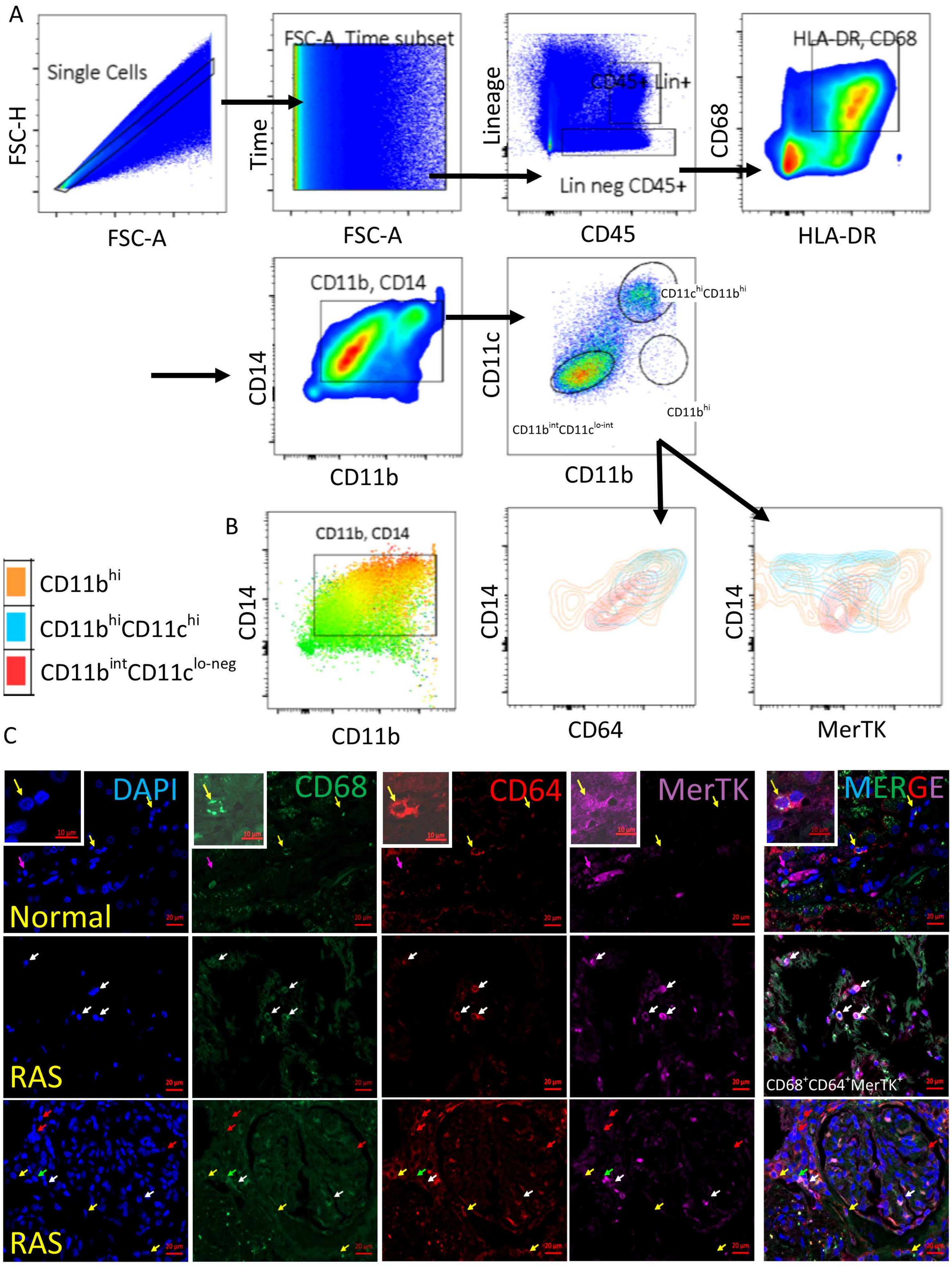

Figure S7. KRM numbers increase in stenotic human kidneys, and inversely correlate with fibrosis.

(A) Flow cytometry of a dissociated non-cancerous portion of a human kidney to identify macrophages. Macrophages in the kidney were identified as Single cells (FSC-H vs. FSC-A), Time Vs. FSC-A, Lineage and Ghost510^{neg}CD45^{+} lymphocytes gated as CD68^{+}HLA-DR^{+}. These macrophages were then gated as CD14^{+}CD11b^{+} followed by CD11b vs CD11c. Like in the murine studies, we identified three populations KRM-like CD11b^{int}CD11c$^{lo\text{-}neg}$, CD11b^{hi}CD11c^{hi}, and CD11b^{hi}. **(B)** CD14 expression increased along with the expression of CD11b. Similarly, expression of CD64 and MerTK was higher in CD11b^{hi}CD11c^{hi} compared to KRM-like CD11b^{int}CD11c$^{lo\text{-}neg}$ population. **(C)** Co-staining (40X) of DAPI, CD68-AF488 (green), CD64-AF594 (red), MerTK AF647 (magenta) and merged images, in healthy and stenotic human kidneys. White arrows indicate cells that show CD68, CD64, and MerTK co-staining; Yellow arrows, co-staining of CD68 and CD64; green arrows: CD68+ cells, magenta: MerTK. Inset image (100X) demonstrates membrane localization of CD64 and MerTK and cytoplasmic CD68.

Supplementary Figure 10.

Top 100 Upregulated Genes in each population with P-value < 0.01 and Fold change > 2.0

Sham KRM vs CD11c^{LO}Mϕ		
Gene ID	log2FC	P.Value
Igha	7.662	0.00194
Mmp12	7.234	0.00000
Igkv1-110	6.730	0.00000
Mmp13	6.653	0.00000
Cadm1	6.189	0.00000
Cd72	6.013	0.00000
Igkv8-21	5.997	0.00130
Ighv5-17	5.943	0.00000
Jchain	5.899	0.00071
Igkv8-19	5.892	0.00002
Iglv1	5.842	0.00026
Srpk3	5.727	0.00000
Scimp	5.655	0.00000
Igkv6-15	5.645	0.00000
Igkv3-2	5.632	0.00042
Ch25h	5.570	0.00000
Slamf9	5.512	0.00000
Ighv1-72	5.499	0.00000
Igkv5-39	5.380	0.00001
Igkv3-4	5.305	0.00024
Ighv10-3	5.264	0.00000
Ighv3-6	5.251	0.00000
Zmynd15	5.198	0.00000
Ighv1-55	5.179	0.00202
Tmem119	5.158	0.00000
Ighv14-2	5.107	0.00000
Igkv4-53	5.096	0.01541
Lilra5	5.091	0.00000
Igkv14-111	5.084	0.00000
Igkc	5.083	0.00044
Igkv4-59	5.076	0.00000
Ighv1-22	5.071	0.00002
Igkv6-23	5.061	0.02926
Igkv10-94	4.977	0.01664
Igkv10-96	4.938	0.01049
Ms4a7	4.924	0.00000
Igkv8-27	4.923	0.02091
Iglc1	4.916	0.00023
Igkv13-84	4.848	0.00025
Igkv14-100	4.834	0.02119
Igkv4-91	4.784	0.00096
Igkv6-20	4.776	0.00017
Cxcl16	4.767	0.00000
Vcam1	4.763	0.00001
Prss30	4.691	0.00000
Cd4	4.691	0.00000
Hpgds	4.603	0.00000
Slco2b1	4.596	0.00000
Igkv6-32	4.581	0.02324
Galnt12	4.564	0.00000
Ighv1-75	4.506	0.00004
Itga9	4.499	0.00000
H2-M2	4.478	0.00000
Lrrc3	4.446	0.00000
Ighv1-26	4.440	0.00087
9430076C15Rik	4.401	0.00000
Igkv1-135	4.391	0.00127
Ighv1-34	4.373	0.00007
Ighv1-76	4.372	0.00012
Col14a1	4.368	0.00000
Asb2	4.366	0.00000
Arhgap22	4.346	0.00000
Ighv1-9	4.346	0.00054
Igsf9	4.310	0.00000
Gm15523	4.309	0.00001
St3gal6	4.303	0.00000
Ighv9-3	4.298	0.02176
Itgax	4.248	0.00000
Cd81	4.246	0.00000
Igkv9-120	4.238	0.00114
Matn2	4.230	0.00001
Nes	4.204	0.00000
Blnk	4.191	0.00003
Igkv1-117	4.171	0.02142
Ptchd1	4.167	0.00000
Axl	4.000	0.00000
Ighv1-52	3.999	0.00013
Fmnl3	3.955	0.00000
Slamf7	3.887	0.00001
Gbp8	3.873	0.00000
Ighv1-18	3.872	0.00010
Tanc2	3.867	0.00000
Fads1	3.867	0.00000
Tnfsf13b	3.867	0.00000
Cttnbp2nl	3.866	0.00000
Igkv4-50	3.862	0.00025
Tgfbr1	3.847	0.00000
Gpr31b	3.775	0.00000
Fcgr4	3.769	0.00000
Gm21860	3.734	0.00081
Gm21748	3.734	0.00081
G530011O06Rik	3.731	0.00000
P3h3	3.705	0.00000
F11r	3.691	0.00000
Serpinf1	3.681	0.00000
Tspan33	3.663	0.00080
Cdk18	3.663	0.00000
Gm20429	3.641	0.00000
Rnf180	3.640	0.00000
Adrb1	3.612	0.000
Dst	3.603	0.000
Smpd5	3.601	0.000
Ms4a14	3.599	0.000
Ighv7-3	3.571	0.027
AU020206	3.564	0.000
Clec9a	3.537	0.000
Ighv1-66	3.535	0.016
Pdgfb	3.534	0.000
Rab3il1	3.533	0.000
Ccnd2	3.533	0.000
Tlr12	3.516	0.000
H2-Eb1	3.484	0.000
1810011H11Rik	3.477	0.000
H2-Ob	3.475	0.000
Dlc1	3.445	0.000
Hs3st3a1	3.445	0.000
Adam33	3.441	0.000
Abcc3	3.439	0.000
A4galt	3.424	0.000
Ighg1	3.414	0.034
Ighv1-54	3.412	0.001
Hs3st1	3.408	0.000
Zfp599	3.404	0.000
C1qa	3.394	0.000
Clec4b1	3.383	0.000
Siglece	3.373	0.000
Meis3	3.370	0.000
Ighv8-8	3.359	0.001
Tgfb3	3.351	0.000
Igkv4-57-1	3.339	0.036
Iglc2	3.333	0.002
Sh3pxd2b	3.317	0.000
Ighv1-78	3.316	0.010
Ighv1-74	3.313	0.021
Itga8	3.306	0.000
Speg	3.291	0.000
Pea15a	3.284	0.000
Hexb	3.275	0.000
Tpbgl	3.263	0.000
Igkv4-74	3.261	0.025
Ets1	3.241	0.000
Sema4c	3.234	0.000
Bend6	3.231	0.000
Cyr61	3.198	0.001
Sytl1	3.192	0.000
Pald1	3.184	0.000
Abca14	3.184	0.000
Gatm	3.174	0.000
Fblim1	3.169	0.000
Hepacam2	3.161	0.000
Cd79b	3.161	0.000
Tle1	3.154	0.000
Cxcr3	3.146	0.000
C1qc	3.145	0.000
Cdk6	3.139	0.000
Tmem171	3.139	0.000
Lacc1	3.132	0.000
Igkv9-123	3.122	0.030
Zfp691	3.115	0.000
Hba-a2	3.087	0.035
C1qb	3.083	0.000
Pvt1	3.073	0.000
Dnase1l3	3.070	0.000
Cd300ld2	3.070	0.000
Rn7sk	3.051	0.042
H2-Ab1	3.044	0.000
Gm15156	3.041	0.000
Igkv4-70	3.040	0.023
Trim47	3.040	0.000
Icosl	3.032	0.000
H2-Aa	2.986	0.000
Ccr5	2.974	0.000
Stab1	2.969	0.000
Mgl2	2.966	0.000
Abhd6	2.951	0.000
C3ar1	2.944	0.000
Gm8818	2.937	0.000
Epor	2.926	0.000
Gpr155	2.926	0.000
Igkv2-137	2.918	0.029
Pmepa1	2.918	0.000
Card11	2.912	0.000
Hbb-bs	2.911	0.027
Itgb5	2.906	0.000
4933424M12Rik	2.901	0.000
Gm4951	2.888	0.000
Plxdc2	2.885	0.000
D8Ertd82e	2.877	0.000
Gadd45g	2.876	0.000
Gm26902	2.875	0.000
Fam214a	2.855	0.000
Cd300ld5	2.854	0.000
P2ry6	2.848	0.000
Sdc4	2.844	0.000
Ccl4	2.841	0.000
Crym	2.837	0.001
Rtnl	2.827	0.000
Tmem221	2.816	0.000
Dtx4	2.800	0.000

Sham KRM vs CD11c^{HI} Mϕ		
Gene ID	log2FC	P.Value
Igkv8-19	5.734	0.00004
Igkv3-4	5.150	0.00040
Igkv5-39	5.091	0.00004
Igkv13-84	4.886	0.00031
Igkv6-32	4.619	0.02489
Ighv9-3	4.162	0.02788
Igkv4-50	3.901	0.00031
Gm21860	3.495	0.01588
Gm21748	3.495	0.01588
Ighg1	3.400	0.03730
Iglc1	3.357	0.01876
Ighv1-78	3.354	0.01036
Igkv9-123	3.160	0.03190
Igkv12-89	3.093	0.03709
Igkv2-137	2.827	0.03681
Cd4	2.784	0.00523
Ighv1-34	2.774	0.03175
Ighv1-52	2.708	0.04366
Iglv1	2.700	0.02987
Ctse	2.688	0.04408
Vcam1	2.542	0.00871
Tmem119	2.487	0.00420
Col14a1	2.459	0.00687
Tpbgl	2.458	0.00285
Ighv4-1	2.436	0.01899
Slco2b1	2.341	0.00296
Gm17147	2.283	0.00084
A4galt	2.263	0.00172
Gm10052	2.256	0.02821
Stab1	2.210	0.00274
Zmynd15	2.200	0.00167
Abcc3	2.198	0.01787
Lrrc3	2.151	0.00248
Cd79b	2.145	0.00572
Adam33	2.097	0.00118
Ighv7-4	2.074	0.03447
Epor	2.048	0.00062
Ptprm	2.037	0.00365
Gm10134	2.023	0.00018
Rnase4	2.011	0.00323
Siglece	2.005	0.00556
Srpk3	2.004	0.00080
C030034L19Rik	1.991	0.006
Ang	1.959	0.003
Gpr31b	1.935	0.009
C3ar1	1.917	0.005
Zfyve28	1.909	0.023
Lilra5	1.909	0.002
Adrb1	1.906	0.007
Pigz	1.890	0.009
Itga8	1.889	0.011
Tnfsf13b	1.881	0.002
Dlc1	1.877	0.005
Meis3	1.855	0.000
Gm15523	1.852	0.000
Itga9	1.841	0.002
Exosc6	1.834	0.019
Abhd15	1.834	0.021
P3h3	1.832	0.005
Cables1	1.830	0.000
Slc40a1	1.829	0.014
Abca14	1.828	0.001
Ms4a7	1.815	0.000
Pla2g15	1.813	0.004
Zfp599	1.806	0.014
Smpd5	1.793	0.001
Igf2bp3	1.778	0.006
Ch25h	1.778	0.000
Gm26520	1.775	0.005
Axl	1.766	0.006
Cd276	1.764	0.006
Mafb	1.757	0.002
Cadm1	1.757	0.001
Cxcl14	1.753	0.002
Olfml3	1.736	0.006
St3gal6	1.734	0.000
Cx3cr1	1.733	0.010
Hs3st1	1.732	0.000
Sepn1	1.729	0.005
C5ar1	1.723	0.007
Rac3	1.722	0.009
Bend6	1.706	0.007
Tmbim1	1.698	0.002
Scimp	1.697	0.003
Arrb2	1.691	0.006
Itgb5	1.689	0.003
RP23-478P10.3	1.679	0.001
C5ar2	1.674	0.007
Lifr	1.668	0.011
P2ry13	1.664	0.013
Gpr65	1.663	0.002
G530011O06Rik	1.661	0.002
Dynlt1c	1.661	0.005
Ighv2-5	1.658	0.021
Pald1	1.657	0.007
Tmem86a	1.656	0.001
Fmnl3	1.653	0.014
Hpgds	1.650	0.000
Ptgs1	1.649	0.002
Cabp4	1.646	0.004
Psd3	1.645	0.003
Gm4951	1.631	0.009
C1qc	1.630	0.002
Tor4a	1.628	0.017
Fblim1	1.625	0.002
Sesn1	1.612	0.007
Galnt12	1.611	0.001
Fut10	1.611	0.012
Loxl3	1.610	0.008
Pydc3	1.608	0.010
Cracr2b	1.608	0.003
Cttnbp2nl	1.599	0.009
Mmp13	1.598	0.001
B3galnt1	1.597	0.003
Hs3st3a1	1.592	0.004
Adgre1	1.591	0.007
C1qa	1.591	0.003
Pmepa1	1.587	0.001
Asb2	1.587	0.001
Ccdc106	1.586	0.003
Zfp691	1.579	0.003
Lair1	1.577	0.011
Slc11a1	1.576	0.015
D130040H23Rik	1.573	0.022
Serpinf1	1.571	0.000
Cyr61	1.570	0.015
Zfp658	1.569	0.001
Tmem191c	1.569	0.001
Gm13710	1.563	0.006
Pydc4	1.563	0.008
Mertk	1.560	0.006
B3gnt8	1.560	0.005
Ddx43	1.560	0.002
1810011H11Rik	1.555	0.004
Hhat	1.553	0.014
Gm12918	1.552	0.020
Gm8818	1.550	0.004
Cd81	1.550	0.001
Gm7805	1.549	0.002
Mylip	1.549	0.007
Ppp1r9a	1.547	0.005
Strip2	1.545	0.028
Tgfbr2	1.545	0.010
Tgfbr1	1.544	0.031
P2ry6	1.539	0.003
Kcnj9	1.539	0.007
Arap3	1.538	0.002
Tanc2	1.532	0.006
Apoe	1.529	0.007
Sh3pxd2b	1.526	0.015
Gm15726	1.522	0.001
Tmem221	1.520	0.001
S1pr2	1.520	0.019
Cxcl16	1.518	0.003
Ntpcr	1.515	0.001
Tlr12	1.512	0.000
Vnn3	1.511	0.024
Xylt2	1.508	0.001
Sema4c	1.506	0.015
Fam214a	1.503	0.003
Pdgfb	1.501	0.001
Gm10382	1.500	0.009
Ccdc142	1.499	0.019
Tmcc3	1.498	0.002
Sap25	1.498	0.000
Dtx4	1.498	0.006
9430076C15Rik	1.498	0.003
Il10rb	1.496	0.004
Gm29291	1.488	0.005
Cdk6	1.488	0.000
Rnf180	1.488	0.001
Ccr5	1.485	0.026
Pfkfb4	1.482	0.008
Gm4955	1.481	0.051
Tlr1	1.481	0.034
Nfxl1	1.479	0.014
Hlx	1.479	0.001
Ptprv	1.471	0.023
Cd72	1.469	0.006
Nes	1.467	0.000
Gm4332	1.464	0.033
1500004A13Rik	1.461	0.004
RP24-502J3.1	1.460	0.010
Chst14	1.452	0.041
Hexb	1.451	0.015
Fcgr4	1.449	0.010
Spsb1	1.448	0.013
Dennd2c	1.445	0.042
Tgfb3	1.442	0.002
Neurl1a	1.441	0.008
Plxnb3	1.440	0.034
Slamf9	1.437	0.010
Oas1g	1.434	0.001
Frmd4b	1.434	0.008
Serinc3	1.429	0.016
Apobec1	1.424	0.006
Cd86	1.421	0.009
Cd14	1.421	0.004
Fcgr1	1.419	0.006

RAS Vs Sham KRM		
Gene ID	lo2gFC	P.Value
Clu	8.459	0.0005
Wfdc2	7.217	0.0004
Klk1	6.914	0.0026
Mgp	6.643	0.0000
Tacstd2	6.566	0.0001
Aqp2	6.554	0.0156
Cldn7	6.212	0.0000
Spp1	6.142	0.0016
Ccl8	6.092	0.0000
Bcam	6.050	0.0002
Krt18	5.998	0.0014
Mal	5.948	0.0000
Hbegf	5.943	0.0002
Tspan8	5.869	0.0000
Umod	5.862	0.0105
Cd24a	5.824	0.0000
Tmem213	5.799	0.0028
Krt8	5.639	0.0041
Hsd11b2	5.638	0.0155
Adamts1	5.617	0.0001
Il1rn	5.594	0.0000
Sfn	5.559	0.0000
Acta2	5.533	0.0004
Scnn1b	5.468	0.0004
Aqp3	5.392	0.0037
Rhcg	5.369	0.0150
Muc1	5.364	0.0010
Crip2	5.312	0.0000
Gstm2	5.288	0.0005
Wfdc15b	5.252	0.0015
Plet1	5.239	0.0046
Bgn	5.227	0.0000
Bhlhe40	5.226	0.0001
Atp6v0d2	5.198	0.0001
Sparc	5.165	0.0000
Elf3	5.146	0.0024
Cdh16	5.120	0.0006
Kng2	5.078	0.0072
Cald1	5.050	0.0000
Ccl7	5.047	0.0000
Lcn2	5.038	0.0065
Defb1	5.014	0.0357
Krt7	4.983	0.0006
Krt19	4.982	0.0000
Clcnkb	4.970	0.0254
Spink8	4.945	0.0136
Tagln	4.942	0.0000
Ctgf	4.940	0.0002
Cldn8	4.906	0.0154
Hoxd8	4.885	0.0004
Tmem52b	4.880	0.0170
Bsnd	4.876	0.0000
Emcn	4.852	0.0000
Fxyd4	4.846	0.0316
Efhd1	4.837	0.0001
Epcam	4.829	0.0091
Pcolce	4.804	0.0000
Sparcl1	4.785	0.0000
Serpinh1	4.764	0.0000
Cldn4	4.734	0.0033
Slc12a1	4.714	0.0265
Cav1	4.681	0.0029
Sytl2	4.677	0.0000
Cd59a	4.675	0.0000
Cxcl13	4.644	0.0000
Tm4sf1	4.640	0.0005
Col18a1	4.636	0.0023
Adgrg1	4.612	0.0081
Atp6v1g3	4.576	0.0170
Kcnj1	4.575	0.0237
Ren1	4.546	0.0035
Rhbg	4.538	0.0129
Spint2	4.533	0.0014
Atp6v1b1	4.511	0.0149
Rasl11a	4.497	0.0012
F3	4.469	0.0007
Slc12a3	4.468	0.0105
Cldn3	4.464	0.0149
Emp1	4.446	0.0000
Ppp1r1a	4.434	0.0006
Rcan2	4.423	0.0000
Rasd1	4.419	0.0151
Tspan1	4.416	0.0172
Apela	4.415	0.0152
Sfrp1	4.410	0.0000
Fermt2	4.387	0.0000
Smtnl2	4.383	0.0002
Tinagl1	4.378	0.0001
Gem	4.372	0.0010
Xcl1	4.357	0.0002
Mal2	4.328	0.0181
Fxyd1	4.318	0.0001
Cdc42ep1	4.313	0.0005
Scnn1g	4.292	0.0124
Scin	4.268	0.0157
Ckmt1	4.256	0.0263
Bicc1	4.252	0.0000
Foxi1	4.234	0.0134
Slc4a9	4.221	0.0125
Serpinb6b	4.210	0.0000
S100a16	4.209	0.0000
Cldn5	4.204	0.0014
Akr1c19	4.183	0.0032
Tspan12	4.182	0.0000
Cdo1	4.177	0.0090
Aif1l	4.153	0.0378
Sdpr	4.145	0.0000
Npnt	4.108	0.0008
Gsta4	4.108	0.0000
Trbc1	4.105	0.0030
Nedd4	4.103	0.0000
Egfl7	4.095	0.0001
Sox4	4.093	0.0000
Tsc22d1	4.082	0.0019
Nkg7	4.081	0.0003
Ptgs2	4.071	0.0006
Paqr5	4.069	0.0155
Slc26a4	4.058	0.0258
Nbl1	4.050	0.0000
F2rl1	4.033	0.0000
Ly6c1	4.029	0.0014
Col1a2	4.007	0.0000
Sprr1a	3.998	0.0121
Dusp5	3.997	0.0000
Atp6v1c2	3.996	0.0121
Wwc1	3.991	0.0001
Col1a1	3.985	0.0000
Slc14a2	3.980	0.0122
Fam174b	3.972	0.0002
Npdc1	3.959	0.0000
Cxcl1	3.957	0.0000
Ighg3	3.943	0.0052
Cpe	3.942	0.0001
Kcnk1	3.941	0.0166
Nr4a2	3.937	0.0000
Col3a1	3.935	0.0000
Fabp4	3.934	0.0006
Il1a	3.915	0.0000
Shisa4	3.899	0.0000
Calb1	3.874	0.0216
Slc4a1	3.870	0.0115
Cd200	3.869	0.0003
Adgrf5	3.869	0.0000
Sostdc1	3.863	0.0310
Cp	3.863	0.0000
Lyz1	3.853	0.0000
Adm	3.849	0.0000
Kcne1	3.834	0.0118
Gm42793	3.833	0.0000
Kcnq1	3.832	0.0122
Gdf15	3.829	0.0000
Spry1	3.817	0.0005
Gprc5a	3.815	0.0131
Il6	3.810	0.0000
Cdh1	3.809	0.0215
Tfcp2l1	3.808	0.0184
Emp2	3.806	0.0000
Id3	3.784	0.0005
Lrrc32	3.772	0.0000
Ntn4	3.769	0.0011
Vegfa	3.752	0.0001
Gpnmb	3.751	0.0027
Il2rb	3.749	0.0002
Ankrd1	3.748	0.0001
Serping1	3.745	0.0000
Nupr1	3.730	0.0000
C3	3.730	0.0000
Mt2	3.726	0.0000
Slc9a3r2	3.721	0.0000
Ptrf	3.720	0.0000
Il10	3.717	0.0000
Phldb2	3.712	0.0000
Stk39	3.712	0.0000
Mcoln3	3.704	0.0000
Ly6a	3.700	0.0053
Ier3	3.680	0.0002
Cryab	3.674	0.0222
Ehf	3.665	0.0000
Atp4a	3.660	0.0176
Car2	3.648	0.0131
Mecom	3.642	0.0002
Aebp1	3.631	0.0000
Serpine2	3.629	0.0003
Aoc1	3.628	0.0112
Ptges	3.620	0.0212
Lmna	3.618	0.0000
Mxra8	3.617	0.0001
S100a10	3.616	0.0000
Rhoj	3.610	0.0007
Trbc2	3.607	0.0003
Aqp1	3.604	0.0008
Id1	3.601	0.0000
Slc35g1	3.601	0.0045
Socs2	3.599	0.0000
Cldn1	3.598	0.0004
4-Sep	3.596	0.0000
Tspan15	3.588	0.0015
Efna1	3.580	0.0028
Rgs5	3.574	0.0004

RAS Vs Sham CD11c^{HI}Mϕ		
Gene ID	log2FC	P.Value
Igkv3-4	7.07	0.00007
Mucl1	5.61	0.00093
Ighv9-3	4.74	0.01241
Igkv6-23	4.56	0.00506
Ccl17	4.47	0.00214
Igkv4-55	4.35	0.04028
Gpnmb	4.13	0.00273
Dcstamp	3.90	0.00076
Cd3g	3.80	0.00042
Ighg2c	3.75	0.00291
Flrt3	3.73	0.02450
Timp1	3.57	0.00047
Trbc2	3.43	0.00188
Trac	3.40	0.00180
Lck	3.28	0.00239
Rps12	3.27	0.00681
Nkg7	3.27	0.00313
Igkv16-104	3.27	0.12141
Trbc1	3.24	0.00087
Nme9	3.18	0.00357
Nr4a3	3.18	0.01483
Cd3e	3.17	0.00085
Iglv2	3.16	0.05885
Tbc1d4	3.15	0.00540
Il7r	3.15	0.00011
Ighg2b	3.14	0.14086
Xcl1	3.12	0.00379
Ighv1-78	3.11	0.12739
Cd6	3.10	0.00001
Tnfrsf4	3.09	0.00025
Klrb1a	3.07	0.00049
Gimap3	3.06	0.00148
Nr4a2	3.04	0.02507
Adam23	3.03	0.00194
Cxcr6	3.02	0.00032
Ctsw	3.01	0.00345
Il2rb	3.00	0.00167
Mgp	2.97	0.01983
Cd3d	2.95	0.00042
Slc27a3	2.94	0.00173
Rpl15-ps3	2.90	0.05317
Cxcl3	2.90	0.00461
Tbx21	2.89	0.00118
Mreg	2.88	0.00340
Gm8113	2.88	0.00027
Il12b	2.87	0.00350
Ighv4-1	2.86	0.12591
Lat	2.86	0.00107
Ighg3	2.85	0.10276
Cd200r3	2.83	0.00077
Pdcd1	2.82	0.00001
Ctsk	2.81	0.01722
Igkv4-57	2.81	0.21125
Cacna1s	2.77	0.00873
Ccl22	2.75	0.00998
Ccr8	2.75	0.00009
AA467197	2.74	0.00320
Gimap4	2.73	0.04022
Hr	2.73	0.00686
Igkv5-39	2.73	0.11819
Igkv9-123	2.71	0.12788
Il1rn	2.70	0.01404
Ccr7	2.69	0.01261
Igkv3-5	2.69	0.16820
Sh2d2a	2.67	0.00008
P2ry10	2.64	0.00730
Tnfrsf9	2.63	0.00001
Areg	2.63	0.02852
Igflr1	2.63	0.03024
Cd5	2.62	0.00001
Gimap7	2.61	0.00033
Gpr171	2.61	0.00686
Ighv1-15	2.61	0.12664
Igkv19-93	2.60	0.27922
Mir155hg	2.60	0.02015
Cd8b1	2.60	0.00015
Igkv4-61	2.55	0.11612
Icos	2.53	0.00007
Wnt11	2.53	0.02543
Rps13-ps2	2.51	0.01536
Gzmb	2.50	0.00873
Gprc5a	2.50	0.02767
Zap70	2.50	0.00021
Cish	2.49	0.00266
Igkv5-45	2.45	0.12954
Kit	2.43	0.00094
Ptprcap	2.43	0.00455
Rpl31-ps8	2.42	0.07166
Col3a1	2.40	0.00591
Tcf7	2.40	0.00067
Il1a	2.39	0.00246
Lrrc32	2.37	0.06755
Ctla4	2.36	0.00132
Procr	2.36	0.00497
Slc9a4	2.36	0.01442
Ighv1-50	2.36	0.20834
Skap1	2.35	0.00612
Ighv11-2	2.31	0.22673
Gfra2	2.31	0.01083
Npy	2.30	0.07724
Gm12918	2.30	0.00833
Aldh1a2	2.26	0.02209
3-Sep	2.25	0.02636
Il1r2	2.25	0.08774
Ighv1-82	2.25	0.24243
Gem	2.25	0.00384
P2rx5	2.24	0.00150
H2-DMb2	2.24	0.01264
Clec4n	2.23	0.00186
Ptgs2	2.23	0.04479
Pxdc1	2.23	0.00842
Mt2	2.23	0.02621
Il4i1	2.20	0.00258
F2rl2	2.20	0.00944
Itk	2.20	0.00014
Plet1	2.19	0.13544
Gpc1	2.16	0.00078
Asgr2	2.16	0.00717
Ear2	2.15	0.03390
Gm11518	2.15	0.02735
Fcrls	2.14	0.01196
Grasp	2.14	0.03767
Mras	2.14	0.00417
Spib	2.14	0.02164
Ppp1r14a	2.14	0.01509
Tnfrsf18	2.13	0.01639
H2-M2	2.13	0.00931
Fscn1	2.13	0.00648
Ifit1bl1	2.11	0.00109
Bcl2a1c	2.11	0.01034
Ighv1-12	2.11	0.16451
Tnfaip8l1	2.11	0.02490
Spink2	2.10	0.01178
E230016K23Rik	2.09	0.00752
H2-Eb2	2.09	0.00550
Cd226	2.08	0.01022
Nccrp1	2.08	0.01864
Cd274	2.07	0.02353
Ngp	2.06	0.13546
Pdcd1lg2	2.06	0.00277
Maff	2.06	0.07602
Pianp	2.05	0.00311
Adam8	2.05	0.00528
Klk8	2.05	0.00198
Il27ra	2.05	0.00212
Trbv3	2.04	0.01140
Fabp5	2.02	0.01179
Ms4a4b	1.99	0.00340
Rpl36a-ps2	1.99	0.00889
Actn1	1.99	0.00720
Fasl	1.98	0.00291
Apol7e	1.98	0.00578
Ltbp3	1.98	0.00618
Izumo1r	1.98	0.00046
Uck2	1.98	0.01561
Mmp2	1.97	0.00695
Cd8a	1.96	0.00028
Irf4	1.96	0.01001
Ighv2-5	1.96	0.13888
Ifitm1	1.96	0.02071
Cd200r1	1.96	0.00693
Ighv6-3	1.95	0.31241
E130012A19Rik	1.95	0.01256
Ffar2	1.95	0.00075
Gm5914	1.94	0.00527
Atp6v0d2	1.94	0.06047
Gm8203	1.93	0.12076
Sh2d1a	1.92	0.00049
Cd40lg	1.91	0.00230
Igkv9-124	1.91	0.28904
Ccl5	1.91	0.00179
Cd80	1.90	0.01918
Il1b	1.89	0.01446
Lum	1.88	0.04358
Hilpda	1.88	0.04890
Rps10-ps2	1.87	0.01587
Klrc1	1.87	0.03297
F7	1.87	0.00260
Cst7	1.86	0.00030
Gm8355	1.86	0.00378
Fosl2	1.85	0.00551
Egr2	1.85	0.00614
Tigit	1.85	0.00019
Crem	1.85	0.05411
Trbv13-1	1.85	0.00009
Anpep	1.85	0.00438
Pacsin1	1.85	0.00365
Igkv12-44	1.85	0.43329
Cyp2s1	1.84	0.01419
Nrp2	1.84	0.00214
Bex6	1.83	0.04987
Col1a2	1.83	0.01810
Gm44135	1.83	0.03654
Sdcbp2	1.83	0.00194
F3	1.83	0.08440
Inhba	1.82	0.03216
Vps37b	1.82	0.02343
Gm15725	1.82	0.00304
Socs2	1.81	0.00190

RAS Vs Sham CD11c^{LO}Mϕ		
Gene ID	log2FC	P.Value
Il23a	3.3372	0.0507
Ccl8	3.3251	0.0047
Mgp	3.2922	0.0346
Acod1	3.0675	0.0484
Mrgpra2b	3.0327	0.0102
Asprv1	2.8958	0.0038
Cd3g	2.7889	0.0003
Stfa2l1	2.7449	0.0226
Arg2	2.6511	0.0466
Mrgpra2a	2.6379	0.0239
Cxcr2	2.6140	0.0104
S100a9	2.5817	0.0125
Igkc	2.5538	0.0331
Vcam1	2.5525	0.0179
Apod	2.4913	0.0161
Inhba	2.4905	0.0183
Il1b	2.4817	0.0021
Il1f9	2.4700	0.0285
Steap4	2.4684	0.0011
S100a8	2.4515	0.0148
Clec4n	2.4435	0.0275
H2-Q10	2.4401	0.0097
Cd83	2.4302	0.0351
Il1rn	2.3877	0.0185
Hdc	2.3641	0.0408
Ms4a7	2.3580	0.0234
Lck	2.3142	0.0028
Nkg7	2.2912	0.0010
Il1bos	2.2607	0.0035
Hpgds	2.2568	0.0245
Flrt3	2.2555	0.0074
Cd101	2.2277	0.0037
Ccl12	2.2103	0.0510
Ctgf	2.1826	0.0390
Gimap3	2.1584	0.0011
Hcar2	2.1584	0.0317
Gimap4	2.1180	0.0003
Scimp	2.1117	0.0148
Gm5483	2.1103	0.0118
Lat	2.1017	0.0012
Iigp1	2.0846	0.0115
Trac	2.0686	0.0108
Mucl1	2.0555	0.0185
Mxra8	2.0449	0.0179
Egfl7	2.0402	0.0444
Bgn	2.0310	0.0296
Slamf7	2.0195	0.0176
Iglc2	2.0057	0.0116
Serpinf1	1.9477	0.0176
Sparcl1	1.9356	0.0281
Ptgs2os2	1.9099	0.0078
Ltf	1.9099	0.0076
Ccnd2	1.8951	0.0102
Trbc1	1.8878	0.0064
Scin	1.8805	0.0092
Dhrs9	1.8637	0.0164
Blnk	1.8543	0.0472
Zmynd15	1.8471	0.0441
Cxcl16	1.8468	0.0483
Cxcr3	1.8374	0.0020
Ralgds	1.8355	0.0472
Slc25a33	1.8184	0.0365
Cd4	1.8143	0.0508
Bcl2a1a	1.7997	0.0184
Fhl2	1.7936	0.0031
Tagln	1.7893	0.0349
Cttnbp2nl	1.7860	0.0178
Il2rb	1.7454	0.0001
Camkk1	1.7250	0.0066
Cp	1.7133	0.0104
St3gal6	1.7057	0.0462
9430076C15Rik	1.6914	0.0203
Tmem171	1.6872	0.0109
Gm15725	1.6791	0.0087
Cxcr6	1.6522	0.0367
H2-Ab1	1.6360	0.0089
Maged2	1.6311	0.0005
Cd3e	1.6281	0.0164
Slamf9	1.6219	0.0476
Olfml3	1.6171	0.0231
H2-Eb1	1.6157	0.0278
P2ry13	1.6110	0.0245
Klrk1	1.6078	0.0123
Rasip1	1.6040	0.0461
Bcl2a1d	1.6030	0.0221
Gm15523	1.5905	0.0421
Ramp2	1.5862	0.0472
Rbpsuh-rs3	1.5846	0.0212
Rabgef1	1.5770	0.0471
Cd274	1.5763	0.0088
Ccr7	1.5762	0.0160
Synpo	1.5713	0.0026
Klrd1	1.5670	0.0004
Rnf180	1.5608	0.0158
Mmp14	1.5535	0.0351
Pxdc1	1.5527	0.0322
Pald1	1.5282	0.0491
Pydc3	1.5266	0.0002
Arl5b	1.5240	0.0333
H2-Aa	1.5192	0.0169
Serpinb6b	1.5191	0.0017
Klra17	1.5087	0.0260

Supplementary Figure 11.

Top 100 Upregulated Genes in each population with P-value < 0.01 and Fold change > 1.5

Sham — KRM vs CD11c^{LO}Mϕ

Gene ID	log2FC	P.Value
Retnlg	-8.6	0.000
Retnla	-7.6	0.000
Alox15	-7.6	0.000
Cxcl13	-7.4	0.000
Fn1	-7.2	0.000
Chil3	-7.2	0.000
Fcna	-7.1	0.000
Hp	-6.8	0.000
Ly6c2	-6.7	0.000
Cd24a	-6.7	0.000
Padi4	-6.7	0.000
F13a1	-6.6	0.000
Sell	-6.5	0.000
Cd177	-6.5	0.000
Saa3	-6.5	0.000
Vsig4	-6.4	0.000
I830127L07Rik	-6.3	0.000
Lyz1	-6.2	0.000
S100a9	-6.2	0.000
Pglyrp1	-6.2	0.000
Mmp9	-6.2	0.000
Ccr3	-6.2	0.000
Plac8	-6.1	0.000
Clec4e	-6.1	0.000
S100a8	-6.0	0.000
F5	-5.9	0.000
Ms4a8a	-5.9	0.000
Mmp8	-5.9	0.000
Pdlim1	-5.8	0.000
Ifitm6	-5.8	0.000
Thbs1	-5.8	0.000
Folr2	-5.8	0.000
Prg4	-5.8	0.000
Plaur	-5.8	0.000
Adam8	-5.7	0.000
S100a4	-5.7	0.000
Glipr2	-5.7	0.000
Lyve1	-5.7	0.000
Slpi	-5.6	0.000
Serpinb2	-5.6	0.000
Nfe2	-5.6	0.000
Lilra6	-5.5	0.000
Capg	-5.5	0.000
Rgcc	-5.5	0.000
Pygl	-5.5	0.000
Cd209f	-5.5	0.000
G0s2	-5.5	0.000
Gm5150	-5.4	0.000
Tppp3	-5.3	0.000
Fam101b	-5.3	0.000
Ccl24	-5.3	0.000
F10	-5.3	0.000
Nfil3	-5.3	0.000
Anxa1	-5.3	0.000
Trem1	-5.3	0.000
Sirpb1c	-5.3	0.000
Rab44	-5.2	0.000
Mcemp1	-5.2	0.000
Hpse	-5.2	0.000
Gda	-5.2	0.000
Atp1a3	-5.2	0.000
Siglecf	-5.2	0.000
Gm9733	-5.2	0.000
Ahnak	-5.2	0.000
Hopx	-5.1	0.000
Gm16194	-5.1	0.000
Il17ra	-5.0	0.000
Fam65b	-5.0	0.000
Arhgef37	-5.0	0.000
Nupr1	-5.0	0.000
S100a6	-4.9	0.000
C3	-4.9	0.000
Wnt11	-4.9	0.000
Cbr2	-4.9	0.000
Rasgrp2	-4.9	0.000
Serpinb1a	-4.9	0.000
Alox5	-4.9	0.000
Serpinb10	-4.9	0.000
Mgst1	-4.9	0.000
Dusp5	-4.8	0.000
Ecm1	-4.8	0.000
S100a10	-4.8	0.000
Inpp5j	-4.8	0.000
Bhlhe40	-4.8	0.000
Rnf125	-4.8	0.000
A530064D06Rik	-4.8	0.000
AI839979	-4.7	0.000
Tuba8	-4.7	0.000
Il4	-4.7	0.000
Apoc2	-4.7	0.000
Rgs18	-4.7	0.000
Cd5l	-4.7	0.000
Tmem154	-4.6	0.000
Fgfr1	-4.6	0.000
Galnt9	-4.6	0.000
Ptgs2	-4.6	0.000
Trem3	-4.6	0.000
Ms4a4a	-4.5	0.000
Chst13	-4.5	0.000

Sham — KRM vs CD11c^{HI}Mϕ

Gene ID	log2FC	P.Value
Retnla	-7.1	0.000
Lyz1	-6.9	0.000
Saa3	-6.0	0.000
Cd209a	-5.6	0.000
Fcna	-5.2	0.000
Fn1	-5.0	0.000
Alox15	-4.9	0.000
Cxcl13	-4.9	0.000
Ear2	-4.7	0.000
Bhlhe40	-4.7	0.000
Ccl24	-4.6	0.000
Prg4	-4.5	0.000
Klrd1	-4.4	0.000
Serpinb2	-4.4	0.000
Ccl17	-4.3	0.000
S100a4	-4.2	0.000
Folr2	-4.2	0.000
Igkv4-68	-4.1	0.030
Mcemp1	-4.1	0.000
Pglyrp1	-4.0	0.002
Ifitm6	-3.9	0.000
Cd24a	-3.9	0.000
Slpi	-3.8	0.000
Igkv4-58	-3.8	0.041
Capg	-3.8	0.000
Fgfr1	-3.8	0.000
Igkv8-24	-3.7	0.030
Cd209f	-3.7	0.000
Clec4e	-3.7	0.000
Ecm1	-3.7	0.000
S100a9	-3.7	0.001
Cd300lg	-3.7	0.000
Igkv12-41	-3.7	0.038
Ahnak	-3.6	0.000
Siglecg	-3.6	0.000
Adam8	-3.6	0.000
Dusp5	-3.6	0.000
S100a10	-3.6	0.000
Padi4	-3.5	0.000
Ms4a8a	-3.5	0.000
Btla	-3.5	0.000
Smim5	-3.5	0.000
Rgs18	-3.5	0.000
Anxa1	-3.5	0.000
Clec10a	-3.5	0.000
S100a6	-3.4	0.000
Plaur	-3.4	0.000
Fcrls	-3.4	0.001
Ccl22	-3.3	0.000
Nupr1	-3.3	0.000
Aldh1a2	-3.3	0.001
Apoc2	-3.3	0.003
Ltb4r1	-3.3	0.001
Cfp	-3.3	0.000
Ly6i	-3.2	0.007
Serpinb1a	-3.2	0.000
Ace	-3.2	0.005
Tppp3	-3.2	0.001
Emp1	-3.2	0.000
Cd300e	-3.2	0.007
S100a8	-3.2	0.001
Tnip3	-3.2	0.000
Plac8	-3.2	0.007
Kcne3	-3.2	0.001
Tgm2	-3.2	0.000
F13a1	-3.2	0.000
Bcam	-3.2	0.001
F10	-3.2	0.000
Cd5l	-3.2	0.002
Cd209d	-3.2	0.000
Gcnt2	-3.1	0.000
Nfil3	-3.1	0.000
Il6	-3.1	0.000
Il17ra	-3.1	0.001
G0s2	-3.1	0.003
Dok2	-3.1	0.001
Hopx	-3.1	0.000
Gramd4	-3.0	0.000
Vsig4	-3.0	0.001
Rgcc	-3.0	0.000
Zbtb46	-3.0	0.000
Tnfsf9	-3.0	0.000
Sirpb1c	-3.0	0.003
Fabp5	-3.0	0.000
Lmo1	-3.0	0.000
Pdlim1	-3.0	0.005
Lyve1	-2.9	0.001
Cbr2	-2.9	0.001
Rasgrp2	-2.9	0.001
Cd209c	-2.9	0.000
Trem1	-2.9	0.009
Hp	-2.9	0.016
Mmp9	-2.9	0.000
Trem3	-2.9	0.013
Ceacam1	-2.9	0.004
Anxa2	-2.9	0.001
Cd209g	-2.8	0.010
Kmo	-2.8	0.000
Gm5150	-2.8	0.002

RAS Vs Sham — KRM

Gene ID	lo2gFC	P.Value
Igkv8-21	-5.7	0.0016
Ighv1-72	-5.3	0.0000
Igkv5-39	-5.2	0.0000
Gm21860	-5.1	0.0000
Gm21748	-5.1	0.0000
Ighv1-9	-5.1	0.0000
Igkv10-96	-4.9	0.0001
Igkv14-100	-4.7	0.0226
Igkv4-53	-4.5	0.0262
Igkv8-19	-4.4	0.0058
Ighv1-75	-4.3	0.0001
Ighv1-34	-4.2	0.0001
Ighv1-55	-4.1	0.0091
Ifnb1	-4.1	0.0093
Ctse	-4.0	0.0000
Ighv14-2	-4.0	0.0002
Ighv1-18	-3.7	0.0002
Igkv4-50	-3.7	0.0004
Igkv6-20	-3.7	0.0040
Gm10052	-3.7	0.0001
Ighv1-52	-3.5	0.0004
Igkv4-57-1	-3.4	0.0283
Ighv10-3	-3.4	0.0163
Igkv3-2	-3.3	0.0239
Ighv1-22	-3.2	0.0030
Prss30	-3.2	0.0025
Igkv13-84	-3.2	0.0504
Ighv1-74	-3.2	0.0251
Ighv1-66	-3.1	0.0288
Ighv3-6	-3.1	0.0161
Cklf	-3.0	0.0432
Galnt12	-3.0	0.0061
Dlc1	-3.0	0.0000
Igha	-2.9	0.0463
1700023H06Rik	-2.9	0.0002
9930111J21Rik1	-2.9	0.0099
Igkv12-89	-2.9	0.0416
B3galt4	-2.9	0.0001
4933424M12Rik	-2.9	0.0001
Flt3	-2.8	0.0000
2010008C14Rik	-2.8	0.0000
Cd4	-2.8	0.0004
Asb2	-2.8	0.0001
Haao	-2.8	0.0013
Dynlt1c	-2.7	0.0033
RP23-296G24.1	-2.7	0.0001
Lifr	-2.7	0.0000
C030034L19Rik	-2.7	0.0000
Igkv4-70	-2.6	0.0380
RP23-218K15.7	-2.6	0.0007
Gm26780	-2.6	0.0001
Gm42688	-2.6	0.0002
Adrb1	-2.6	0.0002
Srpk3	-2.6	0.0001
Gm8696	-2.6	0.0000
Dnase1l1	-2.6	0.0000
Gm44597	-2.6	0.0001
Calhm2	-2.6	0.0000
Gm38140	-2.6	0.0000
Gm15156	-2.6	0.0000
Abhd15	-2.5	0.0001
U2af1l4	-2.5	0.0060
Cabp4	-2.5	0.0000
Gm20605	-2.5	0.0332
Tor4a	-2.5	0.0208
Arrdc2	-2.5	0.0002
Gm21887	-2.5	0.0012
Gm8818	-2.5	0.0005
RP23-394G23.2	-2.5	0.0006
Gm17590	-2.5	0.0004
Slc2a3	-2.4	0.0000
Gm17944	-2.4	0.0001
A330017A19Rik	-2.4	0.0000
Gm38365	-2.4	0.0009
Hspa1b	-2.4	0.0012
Snhg17	-2.4	0.0036
Hrh1	-2.4	0.0000
Fkbp5	-2.4	0.0001
Igf2bp3	-2.4	0.0000
Gm15513	-2.4	0.0000
Gm42660	-2.4	0.0024
B3gnt8	-2.4	0.0003
Fut7	-2.4	0.0002
Icosl	-2.4	0.0038
Gm16712	-2.4	0.0003
Il16	-2.4	0.0001
Hist1h2bg	-2.4	0.0017
1700084C06Rik	-2.3	0.0001
Zfp276	-2.3	0.0009
Tlr4	-2.3	0.0184
Ighv2-6	-2.3	0.0167
Gm17147	-2.3	0.0001
Rad9a	-2.3	0.0006
Slco2b1	-2.3	0.0020
Pisd-ps1	-2.3	0.0367
Rac3	-2.3	0.0124
Rwdd2a	-2.3	0.0000
AI467606	-2.3	0.0000
Hist2h2ac	-2.3	0.0009

RAS Vs Sham — CD11c^{HI}Mϕ

Gene ID	log2FC	P.Value
Igkv6-15	-5.22	0.01294
Alox15	-4.45	0.01549
Prg4	-4.03	0.00191
Igkv4-58	-3.81	0.03668
Cd209f	-3.76	0.00529
Mmp13	-3.72	0.00842
Ighv1-9	-3.58	0.02255
Srpk3	-3.49	0.00727
Slpi	-3.31	0.01313
Ifnb1	-3.19	0.02980
Lilra5	-3.18	0.03320
Fcna	-3.05	0.02424
Padi4	-2.87	0.02518
Itga9	-2.85	0.02301
Cd209g	-2.81	0.01440
Cbr2	-2.76	0.03969
Folr2	-2.58	0.03501
Mtmr11	-2.57	0.00676
Tstd1	-2.50	0.03518
Ch25h	-2.45	0.01700
Lyve1	-2.41	0.04259
Crym	-2.26	0.01046
Agmo	-2.26	0.00925
Cd300ld2	-2.25	0.00483
Gm15931	-2.15	0.02489
Gjb2	-2.11	0.04421
Serpinb2	-2.10	0.03820
St3gal6	-2.07	0.01701
G530011O06R	-2.05	0.00588
Ms4a14	-2.01	0.02152
Mir99ahg	-1.98	0.02910
Hs3st1	-1.96	0.02629
Gm15523	-1.95	0.03532
Tanc2	-1.94	0.04086
Spaca6	-1.94	0.03778
Hspa1b	-1.93	0.03756
Hpgd	-1.93	0.05087
Gm8696	-1.92	0.01499
Gstp2	-1.92	0.00570
F5	-1.91	0.02120
Ophn1	-1.90	0.02852
Trpv4	-1.86	0.01376
Hspb1	-1.85	0.03302
Ednrb	-1.84	0.01090
B3galnt1	-1.84	0.01998
Dlc1	-1.83	0.03304
Trim47	-1.80	0.02533
Qprt	-1.80	0.02008
Arhgef10l	-1.77	0.02562
Ltc4s	-1.76	0.04414
Arvcf	-1.76	0.01998
Slc22a17	-1.75	0.03845
Cdk6	-1.75	0.02461
Gm16104	-1.75	0.00075
Snx29	-1.75	0.02929
Rny3	-1.72	0.03598
RP23-296G24	-1.71	0.00825
Ighv1-39	-1.69	0.04436
Hspa1a	-1.68	0.04372
Siglech	-1.66	0.02549
Matn2	-1.64	0.01158
Sh3bp5	-1.64	0.02793
Gsr	-1.63	0.03346
C2	-1.62	0.04966
Psd3	-1.62	0.04667
Pgpep1	-1.62	0.04810
Hes1	-1.61	0.03869
Gm8818	-1.61	0.02109
Dst	-1.61	0.01523
Arap3	-1.60	0.03915
Pou6f1	-1.60	0.00829
Slc7a8	-1.59	0.04105
Rhbdf1	-1.59	0.01945
Gm5129	-1.58	0.00372
Klhl13	-1.56	0.02457
Itga6	-1.56	0.03940
Slc12a2	-1.55	0.01757
Gcnt1	-1.54	0.05088
Cyr61	-1.54	0.01902
Sephs2	-1.54	0.04532
Gm14117	-1.53	0.01627
D8Ertd82e	-1.53	0.02571
Ccdc141	-1.50	0.05026
Prtn3	-1.50	0.00128

RAS Vs Sham — CD11c^{LO}Mϕ

Gene ID	log2FC	P.Value
Cyp4a12a	-2.3952	0.0442
Gm25099	-1.9450	0.0000
Gm24497	-1.9081	0.0455
Gm12854	-1.8478	0.0075
Hal	-1.7939	0.0145
I830127L07Ri	-1.7582	0.0376
Gpm6a	-1.7234	0.0425
Snora31	-1.6777	0.0046
BC025446	-1.6466	0.0491
Acnat2	-1.6432	0.0278
RP23-394G23.	-1.6153	0.0426
BC089597	-1.5468	0.0498
Selenbp2	-1.5111	0.0430
Gm20658	-1.5015	0.0482

1.1. Tissue digestion, single cell preparation, and flow cytometry

After perfusion with ice-cold HBSS, harvested kidneys were diced and digested with 0.05 mg/ml LiberaseTL (Millipore-Sigma) and 100U/ml DNase (Thermo Fischer Scientific)[1]. Addition of RMPI media with 10% FBS was followed by filtration of the suspension through a cell strainer. The filtrate was then centrifuged at 300*g* for 10 min and the pellet resuspended as single cells. Lymphocytes from single cells were separated using Percoll gradient (70:30) (Sigma) and stained with Live-Dead stain for 15min at 10℃. Then the cells were blocked with FCR block (Miltenyi Biotec) for 15 min at 10℃ and stained with antibody cocktail as stated in Supp Table 3 and 4 for one hour at 10℃.

For flow sorting, cell were washed and suspended in MACS buffer (Miltenyi Biotech) and immediately sorted using 4-laser Aria (BDBiosciences). Lineage positive cells (T-cells, B-cells, NK cells, erythrocytes and granulocytes) were gated out using a dump channel. Macrophages in table 1, were flow sorted directly in RNA lysis buffer. Cell digestion to cell sorting in lysis bugger took around 5.5 hours.

For flow cytometry analysis, cells were then fixed with Fix and Lyse (eBioscience) for 5min, washed twice with flow buffer, and acquired using Fortessa X20 Around 5 million events were collected and exported as FCS3.0 and analyzed with FlowJo (FlowJo LLC). The percent of live and lineage negative cells, median fluorescence intensity of CD64 or F4/80 (MFI), and robust standard deviation (rSD) were calculated. Macrophage expression was measured as resolution metrics (Rd), calculated as MFI(experimental)-MFI(control)/rSD(experimental)+rSD(control), where rSD stands for robust standard deviation (rSD)[2]. The reason of normalizing the data using resolution metric is that these experiments are performed multiple times using flow cytometry. Therefore, it was best to convert the median fluorescence intensity data to a fold over background, or resolution metric (RD) value. The RD is better as it accounts for the spread of the data, not just the separation between experimental and control. The use of RD has been described in depth at https://expertcytometry.com/flow-cytometry-statistics/

Anti-Mouse and Anti-Rat Compensation beads plus (BDBiosciences) and one-comp ebeads (eBioscience) were used to create compensation controls. All antibodies were titrated and fluorescence-minus-one control (FMO) was used to set gates.

1.2. In vivo macrophage depletion studies

To create BM-chimeric mice, wild-type CD45.2 mice (n=20) were irradiated with 1100 rads. Within 24 hours, mice were injected retro-orbitally with a minimum of 200,000 BM cells isolated from five CD45.1 (donor) mice and were subsequently maintained on enrofloxacin (Baytril, 22 mg/Kg) added in drinking water. At 8 weeks, these mice underwent RAS or Sham (n=10 each) surgeries. Four weeks later, mice were euthanized, and kidneys harvested for flow cytometry, stored in formalin, or snap frozen.

In subsets of RAS (n=10/group) and sham (n=10/group) mice, a low-dose of liposomal clodronate 100ul (FormuMax Scientific CA) or vehicle (empty liposomes) was injected intraperitoneally every 4 days for 4 weeks[3,4].

1.3. RNA sequencing

For RNA-seq, cells were prepared and stained with antibodies, washed with flow buffer, and subjected to flow sorter (BD Aria III). F4/80^{+}CD64^{+}macrophages from CD11c^{hi}, CD11c^{lo} and KRM and CD45^{+}CD11b/c^{neg} (Figure 1A-C) were sorted (n=2-5 samples/group) in RNA

isolation lysis buffer (Qiagen Inc.; see Flow sorting strategy in Figure 1). RNA-Seq was carried out using v4 Ultra Low-Input RT kit from Clontech (Catalogue#634889) followed by Nextera XT Library Preparation Kit from Illumina (FC-131-1024). RNA-Seq paired-end reads were aligned to the mm10 mouse genome using OmicSoft software's OSA aligner, and genes were annotated using Ensembl.R83. Estimated gene counts were Transcripts Per Million (TPM) scaled and quantile normalized. Pairwise comparisons between macrophage populations ($CD11c^{hi}M\phi$, $CD11c^{lo}M\phi$, and KRM), as well as comparisons between sham and RAS for each macrophage population, were conducted by applying Wald test of the negative binomial distribution to the log2 gene counts using the DESeq2 statistical package[5], and genes that showed statistically significant differences were selected (fold-change>2, P<0.05).

For enrichment analysis of biological process ontology, differentially expressed genes were analyzed in DAVID[6,7] and processes were selected based on P <0.05. Sham KRM n=4, Sham $CD11c^{hi}M\phi$, n=3, Sham $CD11c^{lo}M\phi$, n=3; RAS KRM n=3, RAS $CD11c^{hi}M\phi$, n=3 and RAS $CD11c^{lo}M\phi$, n=3. Differentially expressed genes and pathway analysis has been deposited in GEO (#GSE116094).

1.4. Gene expression and TaqMan low-density array (TLDA)

For validation of RNA-seq studies, RNA was isolated using PureLink® RNA Micro Scale Kit (Thermo Scientific) within a week of sample collection. Quantification was carried using Nanodrop (Thermo Scientific), and approximately 50ng of RNA reverse transcribed to cDNA using Maxima First Strand cDNA Synthesis Kit for qPCR (Thermo Scientific). TLDA plates were custom ordered for genes of interest selected from module 161 of Immgen[8-10]. *Actb, Hprt, Gusb* and *18S* were used as reference genes. After pre-amplifying cDNA using custom cocktail master mix, the amplified product used for TLDA on Viia7 (Thermo Scientific). The cycle threshold values (Ct) of target genes were normalized to the geometric mean of Ct values of reference genes. Gene expression was calculated as delta-delta Ct and plotted as log values of expression (Figure S1E, S5E, S8G, H and 4E). n=4 samples per RAS and Sham group.

1.5. Mouse Kidney Fibrosis Assessment

Paraffin-embedded mid-hilar renal cross-sections (5µm thick) were stained with hematoxylin and eosin (H&E), trichrome, or picro-Sirius Red to study renal fibrosis and peri-tubular endothelial cells. Staining was semi-automatically quantified in 10-15 fields per slide at 40X and 100X using AxioVision (Carl Zeiss MicroImaging, Thornwood, NY), and expressed as the fraction of kidney surface area. Peritubular endothelial cells in Sham, RAS, BMT+Sham and BMT+RAS (n=5 per group) mice kidneys were identified at 100X. These cells were identified as peritubular cells that have lumen, nucleus and a red blood cell as described previously[11 12]. These cells were then quantified at 100X. Results from all fields were averaged.

1.6. Imaging Mouse Kidney Peri-tubular endothelial cells by immunofluorescence

As previously described, PLVAP+CD31+ cells were identified as peri-tubular endothelial cells[13-1516]. Sham and RAS kidneys (n=6 per group) were frozen in OCT and 10µm sections stained for CD31 (AF647) and PLVAP (AF488) (Biolegend). In the peri-tubular region of kidney CD31+PLVAP+ cells were identified. These cells were counted per 40X field and 10 such fields were counted per sample and averaged. Images were captured at 40X using Zeiss Apotome fluorescent microscope and Zen imaging software[17].

For mice in the BMT groups, formalin-fixed paraffin-embedded kidneys of BMT+Sham and BMT+RAS mice were sectioned (5 um), deparaffinized, and incubated sequentially with PLVAP (Novus) and CD31 (Cell Signaling) antibodies, followed by Opal polymer HRP (anti-mouse and anti-rabbit Ig cocktail; Perkin Elmer), and then developed with tyramide signal amplification (TSA)-conjugated 620 and 690 Opal fluorophores (PerkinElmer), according to the manufacturer's instructions. Slides were incubated in PerkinElmer AR9 buffer for both the heat-induced epitope retrieval as well as antibody stripping after each staining cycle. Sections were imaged on a Vectra3 multispectral imaging system. Spectral unmixing, auto-fluorescence elimination and cell quantitation were all performed using InForm software[18].

1.7. In vitro experiments

Murine embryonic fibroblasts (MEF) were generated from GFP+ embryos (n=3) of Col1a1-GFP mice[19] in Advanced DMEM (Thermo Fischer Scientific) with 1%FBS (ATCC). At passage 1, MEF were co-incubated with or without macrophage subpopulation flow-sorted from kidneys of 10-week-old mice in Advanced RPMI with 1% serum and 1ng of M-CSF (Peprotech). Kidney-resident macrophages from Sham and RAS kidneys, $CD11c^{hi/lo}M\phi$ and bone marrow-derived macrophages were flow-sorted and labeled with the anti-mouse CD64-AF647 (Biolegend) at a ratio of 4:1 in a 6-well plate (Figure 5D). In addition, TGF-β (Peprotech) was added to MEFs or co-cultures at doses of 0.5, 1 or 2ng/ml for 18 hours in serum-free condition. Then cells were trypsinized by TrypLE™ Express, washed, and stained with Hoechst 33342 (1ng/ml) (Thermo Fischer Scientific). Cells were acquired using FlowSight (Millipore), mean pixel intensity and mean absolute standard deviation of GFP were measured from $CD64^{neg}Hoechst^{+}$ (nucleated) cells using Ideas® (Millipore), and Rd calculated. P-value of the data was measured by using Mann-Whitney test. The dependence on TGF-β signaling was confirmed using UO126 (MEK pathway inhibitor) and LY2109761 (TGF-β receptor inhibitor). BMϕ = bone marrow macrophages; Mϕ1, 2 are $CD11c^{hi/lo}M\phi$ (n=5 technical replicates and n=3 biological replicates per sample);

To identify the effect of KRM on proliferation of endothelial cells, $PLVAP^{+}CD31^{+}Ly6c^{-}$ renal peri-tubular endothelial cells were flow-sorted from 10-day old C57 mice (n=10) and co-cultured with RAS and Sham KRM. Proliferation was measured by EdU incorporation and Cell Trace IR (Thermo Fischer # C10424). Foxo1 inhibitor (AS1842856, Calbiochem) was used as control for EdU incorporation studies. Inhibition of FOXO1 has been shown to enhance angiogenesis in capillaries, resulting in microvascular regeneration and improved function in mouse models of injury-repair[20].

1.8. Patient Protocol:

Patients were identified as part of a clinical investigation of tissue oxygenation in human renovascular disease between 2008 and 2012. Fourteen patients underwent trans-venous biopsy of the right-sided stenotic kidney via the jugular vein. Inclusion criteria were the presence of unilateral right-sided ARAS >70% obstruction, as previously described[21], and systolic hypertension >155 mmHg, and/or the use at least of two antihypertensive medications (Table 1). Diabetic patients were excluded, as were patients with serum creatinine >2.0 mg/dl. Informed, written consent was obtained after receiving approval from the Institutional Review Board of the Mayo Clinic in adherence with the Declaration of Helsinki. All patients were treated with either an angiotensin converting enzyme inhibitor or angiotensin receptor blocker for hypertension. A

3-day inpatient protocol was performed in the Clinical Research Unit of St. Mary's Hospital, Rochester, Minnesota. Daily isocaloric sodium intake was maintained at 150 mEq.

For the healthy group, implantation biopsies obtained from 15 living kidney donors, selected to have a similar distribution of age and sex, were identified from the Mayo Kidney transplant program as previously described [22]. Detailed analysis of interstitial, glomerular, and vascular compartments for each sample was performed by a senior renal pathologist blinded to the sample source. Hematoxylin and eosin, periodic acid–Schiff, and Masson's trichrome stains were employed. Banff '97 grading systems were used to assign scores for interstitial, glomerular, and vascular lesions. Each sample was graded for interstitial fibrosis, inflammation, and vascular changes [22 23]. The degree of interstitial fibrosis and inflammation was scored according to the estimated fraction of affected renal parenchyma: 0 corresponded to <25%; 1, 25%–50%; 2, 51%–75%; and 3, >75% of tissue affected [24].

1.9. Hemodynamic data for RAS patients:

Patients with unilateral RAS were studied during a 3-day inpatient protocol as reported previously [24]. In brief, the first study day included measurement of GFR by iothalamate clearance. Blood pressure was measured by automated oscillometric recordings at 4-hour intervals. On the third day of the protocol, the right internal jugular vein was cannulated with a 6F sheath and blood samples were drawn from the right and left renal veins and infrarenal inferior vena cava with a 5F pigtail Cobra catheter (Cook Inc, Bloomington, IN) for venous oxygen levels. The catheter was then replaced with a 5F pigtail, which was placed into the superior vena cava for central venous injection of contrast for transit time studies using a multidetector computed tomography (MDCT). Image analysis was performed using ANALYZE (Biomedical Imaging Resource Center, Mayo Clinic, Rochester, MN). Analysis of MDCT flow studies was undertaken by selecting regions of interest in cross-sectional images from the aorta, individual kidney cortex, and medulla. Single-kidney blood flows were determined as the sum of medullary and cortical blood flows, defined by medullary and cortical perfusion per cubic centimeter of renal tissue and volumes calculated using the stereology module within ANALYZE. Single kidney-GFR was determined by apportioning the measured Iothalamate clearance by percentage of blood flow for each kidney[25]. After completion of MDCT studies, the jugular vein access sheath was upsized to 9F and patients underwent biopsy of the right kidney using a transjugular biopsy set (Cook Inc)[22].

1.10. Immunofluorescence labeling of human kidney biopsies:

For detection of KRM, fluorescence-conjugated antibodies against the following proteins were used for immuno-labeling: CD64 (1:100, Clone 10.1; Abcam), MerTK (1:50, Clone Polyclonal; Thermo Scientific), CD11b-AF594 (1:100, Clone M1/70; Biolegend), CD11c-AF647 (1:100; Clone HC1/1 Novus Biologics), CD68-AF488 (1:100 Clone FA-11; Abcam) and co-labelled with ProLong Gold/DAPI. N=5-7 images per sample were acquired at 40X and 100X using Apotome imaging system (Zeiss) and processed using Zen 2.0 software (Zeiss). The total number of cells per slide unit area was determined and plotted. CD11b/c high and low expression was identified by increasing the exposure. Cells expressing high levels of CD11b/c would saturate while the cells expressing low levels of CD11b/c would not. We measured fluorescence of CD11b/c high cells. In general the pixel intensity of CD11b/c^{int} cells was estimated as 0.5 to 0.7 of the pixel intensity of CD11b/c high cells. The exposure was kept constant throughout the sample.

Non-tumor pieces of kidneys were obtained from patients undergoing nephrectomy for renal-cell carcinoma (IRB#16-009485). These relatively healthy kidney pieces were enzymatically digested and subjected to flow cytometry to identify macrophage markers (Table S3). Informed, written consent was obtained after receiving approval from the Institutional Review Board of the Mayo Clinic in adherence with the Declaration of Helsinki from all patients.

1 Kawakami, T. *et al.* Resident renal mononuclear phagocytes comprise five discrete populations with distinct phenotypes and functions. *J Immunol* **191**, 3358-3372, doi:10.4049/jimmunol.1300342 (2013).

2 Zucker, R. M., Ortenzio, J. N. & Boyes, W. K. Characterization, detection, and counting of metal nanoparticles using flow cytometry. *Cytometry A* **89**, 169-183, doi:10.1002/cyto.a.22793 (2016).

3 Li, Z., Xu, X., Feng, X. & Murphy, P. M. The Macrophage-depleting Agent Clodronate Promotes Durable Hematopoietic Chimerism and Donor-specific Skin Allograft Tolerance in Mice. *Scientific reports* **6**, 22143, doi:10.1038/srep22143 (2016).

4 Winkler, I. G. *et al.* Bone marrow macrophages maintain hematopoietic stem cell (HSC) niches and their depletion mobilizes HSCs. *Blood* **116**, 4815-4828, doi:10.1182/blood-2009-11-253534 (2010).

5 Love, M. I., Huber, W. & Anders, S. Moderated estimation of fold change and dispersion for RNA-seq data with DESeq2. *Genome Biol* **15**, 550, doi:10.1186/s13059-014-0550-8 (2014).

6 Huang, D. W., Sherman, B. T. & Lempicki, R. A. Systematic and integrative analysis of large gene lists using DAVID bioinformatics resources. *Nature protocols* **4**, 44-57, doi:10.1038/nprot.2008.211 (2009).

7 Huang, D. W., Sherman, B. T. & Lempicki, R. A. Bioinformatics enrichment tools: paths toward the comprehensive functional analysis of large gene lists. *Nucleic Acids Research* **37**, 1-13, doi:10.1093/nar/gkn923 (2009).

8 Gautier, E. L. *et al.* Gene-expression profiles and transcriptional regulatory pathways that underlie the identity and diversity of mouse tissue macrophages. *Nature immunology* **13**, 1118-1128, doi:10.1038/ni.2419 (2012).

9 Shay, T. & Kang, J. Immunological Genome Project and systems immunology. *Trends Immunol* **34**, 602-609, doi:10.1016/j.it.2013.03.004 (2013).

10 Benoist, C., Lanier, L., Merad, M., Mathis, D. & Immunological Genome, P. Consortium biology in immunology: the perspective from the Immunological Genome Project. *Nat Rev Immunol* **12**, 734-740, doi:10.1038/nri3300 (2012).

11 Ebrahimi, B. *et al.* Addition of endothelial progenitor cells to renal revascularization restores medullary tubular oxygen consumption in swine renal artery stenosis. *Am J Physiol Renal Physiol* **302**, F1478-1485, doi:10.1152/ajprenal.00563.2011 (2012).

12 Sun, D. *et al.* Experimental coronary artery stenosis accelerates kidney damage in renovascular hypertensive swine. *Kidney international* **87**, 719-727, doi:10.1038/ki.2014.343 (2015).

13 de Bruin, R. G. *et al.* The RNA-binding protein quaking maintains endothelial barrier function and affects VE-cadherin and beta-catenin protein expression. *Scientific reports* **6**, 21643, doi:10.1038/srep21643 (2016).

14 Herrnberger, L. *et al.* Formation of fenestrae in murine liver sinusoids depends on plasmalemma vesicle-associated protein and is required for lipoprotein passage. *PloS one* **9**, e115005, doi:10.1371/journal.pone.0115005 (2014).

15 Herrnberger, L., Ebner, K., Junglas, B. & Tamm, E. R. The role of plasmalemma vesicle-associated protein (PLVAP) in endothelial cells of Schlemm's canal and ocular capillaries. *Experimental eye research* **105**, 27-33, doi:10.1016/j.exer.2012.09.011 (2012).
16 Imberti, B., Morigi, M. & Benigni, A. Potential of mesenchymal stem cells in the repair of tubular injury. *Kidney Int Suppl (2011)* **1**, 90-93, doi:10.1038/kisup.2011.21 (2011).
17 Eirin, A. *et al.* Adipose tissue-derived mesenchymal stem cells improve revascularization outcomes to restore renal function in swine atherosclerotic renal artery stenosis. *Stem cells* **30**, 1030-1041, doi:10.1002/stem.1047 (2012).
18 Parra, E. R. *et al.* Validation of multiplex immunofluorescence panels using multispectral microscopy for immune-profiling of formalin-fixed and paraffin-embedded human tumor tissues. *Scientific reports* **7**, 13380, doi:10.1038/s41598-017-13942-8 (2017).
19 Magness, S. T., Bataller, R., Yang, L. & Brenner, D. A. A dual reporter gene transgenic mouse demonstrates heterogeneity in hepatic fibrogenic cell populations. *Hepatology* **40**, 1151-1159, doi:10.1002/hep.20427 (2004).
20 Dang, L. T. H. *et al.* Hyperactive FOXO1 results in lack of tip stalk identity and deficient microvascular regeneration during kidney injury. *Biomaterials* **141**, 314-329, doi:http://dx.doi.org/10.1016/j.biomaterials.2017.07.010 (2017).
21 Gloviczki, M. L. *et al.* Preserved oxygenation despite reduced blood flow in poststenotic kidneys in human atherosclerotic renal artery stenosis. *Hypertension* **55**, 961-966, doi:10.1161/HYPERTENSIONAHA.109.145227 (2010).
22 Rea, D. J. *et al.* Glomerular volume and renal histology in obese and non-obese living kidney donors. *Kidney international* **70**, 1636-1641, doi:10.1038/sj.ki.5001799 (2006).
23 Racusen, L. C. *et al.* The Banff 97 working classification of renal allograft pathology. *Kidney international* **55**, 713-723, doi:10.1046/j.1523-1755.1999.00299.x (1999).
24 Gloviczki, M. L. *et al.* TGF expression and macrophage accumulation in atherosclerotic renal artery stenosis. *Clin J Am Soc Nephrol* **8**, 546-553, doi:10.2215/CJN.06460612 (2013).
25 Saad, A. *et al.* Stent revascularization restores cortical blood flow and reverses tissue hypoxia in atherosclerotic renal artery stenosis but fails to reverse inflammatory pathways or glomerular filtration rate. *Circulation. Cardiovascular interventions* **6**, 428-435, doi:10.1161/CIRCINTERVENTIONS.113.000219 (2013).

Supplementary Table 1. Characteristics of patients with renal artery stenosis

	**Renal Artery Stenosis (n=14)**
Age (y)	66.9±9.0
Sex (men:women)	9:5
Body mass index (kg/m^2)	26.6±4.03
Systolic blood pressure (mmHg)	141.6±18.2
Diastolic blood pressure (mmHg)	71.1±7.4
Duration of Disease (months)	16±16
Urinary micro albumin (mg/24H)	19.7±16.8
Serum creatinine (mg/dL)	1.2±0.5
Single Kidney GFR	27.97±17.8
Total cholesterol (mg/dl)	173.2±33.6
High-density lipoprotein (mg/dl)	46.0±17.06
Low-density lipoprotein (mg/dl)	98.5±25.8
Triglycerides (mg/dl)	143.0±83.4
**Medications (number of patients):**	
Angiotensin receptor blocker	6/14
Angiotensin-converting enzyme inhibitor	10/14
Calcium-channel blocker	10/14
Diuretics	6/14
α-Blockers	2/14
Statins	6/14

Data are Mean±Standard deviation (SD), GFR, glomerular filtration rate

Supplementary Table 2: Antibodies, dilutions and reagents used in mouse flow cytometry experiments.

Antibody	Clone	Flurophore	Dilution (Volume in ul/100ul/10^7cells)	Source
Ly6G	1A8	BUV396	2	BD Biosciences
CD43	S7	BUV737	3	BD Biosciences
CD64 (FcγR-1)	X54-5/7.1	BV421	3	Biolegend
CD43	S7	BV510	4	BD Biosciences
Ghost		510	0.5	Tonbo Biosciences
CD45.1	A20	BV605	2	Biolegend
Cx3cr1	SA011F11	BV650	2	Biolegend
CD11b	M1/70	BV711	4	BD Biosciences
Ly6c	HK1.4	BV786	0.5	Biolegend
Zombie Green			0.5	Biolegend
FCRIV		PE and PEcy7	2	Biolegend
CD3e, CD19, Ter119, NK1.1, Ly6G	1D3/CD19, 145-2C11, TER-119, PK136, IA8	FITC or APCcy7 or BUV395	4ul<br><br>Each	Tonbo/Biolegend/BD Biosciences
MHCII	M5/114.15.2	Percp-eF710	2	eBioscience
MerTK	Polyclonal Goat	PE or APC	7	R and D systems
MerTK	DS5MMER	PE	6	eBioscience
F4/80	BM8	Pe-cy7	4	Biolegend
CD11c	HL3	PECF594 or APCR700	5	BD Biosciences
CD206	C068C2	APC	6	Biolegend
CD45.2	104	APCeF780 or FITC	3	eBioscience
CD45	30F11	APCVio770	6	Miltenyi Biotec
CD26		PEVio770	5	Miltenyi Biotec
PLVAP	MECA32	AF488	5	Biolegend
EdU		AF647	3	Thermo Fischer
BrdU		AF647	5	Thermo Fischer
CD31		BUV395 or BV605	3	BD Bioscience

Supplementary Table 3: Human antibodies, dilutions and reagents used in flow cytometry experiments.

Antibody	Clone	Flurophore	Dilution (Volume in ul/100ul/10^7cells)	Source
Ly6G	1A8	BUV396	2	BD Biosciences
CD14	S7	BUV737	3	BD Biosciences
CD64 (FcγR-1)	X54-5/7.1	BV421	3	Biolegend
CD16	S7	BV510	4	BD Biosciences
Ghost		510	0.5	Tonbo Biosciences
CD45	A20	BV605	2	Biolegend
Cx3cr1	SA011F11	BV650	2	Biolegend
CD11b	M1/70	BV711	4	BD Biosciences
FCRIV		PE and PEcy7	2	Biolegend
CD3e, CD19, Ter119, NK1.1, Ly6G	1D3/CD19, 145-2C11, TER-119, PK136, IA8	FITC or APCcy7 or BUV395	4ul Each	Tonbo/Biolegend/BD Biosciences
HLA-DR	M5/114.15.2	Percp-eF710	2	eBioscience
MerTK	Polyclonal Goat	PE or APC	7	R and D systems
CD68	BM8	Pe-cy7	4	Biolegend
CD11c	HL3	PECF594 or APCR700	5	BD Biosciences
CD206	C068C2	APC	6	Biolegend
CD45	30F11	APCVio770	6	Miltenyi Biotec
CD31		PEVio770	5	Miltenyi Biotec
CD31		BUV395 or BV605	3	BD Bioscience
